# Context specificity of the EMT transcriptional response

**DOI:** 10.1038/s41467-020-16066-2

**Published:** 2020-05-01

**Authors:** David P. Cook, Barbara C. Vanderhyden

**Affiliations:** 1 Cancer Therapeutics Program, Ottawa Hospital Research Institute, Ottawa, ON Canada; 2 Department of Cellular and Molecular Medicine, University of Ottawa, Ottawa, ON Canada

**Keywords:** Metastasis, Tumour heterogeneity, High-throughput screening

## Abstract

Epithelial–mesenchymal plasticity contributes to many biological processes, including tumor progression. Various epithelial–mesenchymal transition (EMT) responses have been reported and no common, EMT-defining gene expression program has been identified. Here, we have performed a comparative analysis of the EMT response, leveraging highly multiplexed single-cell RNA sequencing (scRNA-seq) to measure expression profiles of 103,999 cells from 960 samples, comprising 12 EMT time course experiments and independent kinase inhibitor screens for each. We demonstrate that the EMT is vastly context specific, with an average of only 22% of response genes being shared between any two conditions, and over half of all response genes were restricted to 1–2 time course experiments. Further, kinase inhibitor screens revealed signaling dependencies and modularity of these responses. These findings suggest that the EMT is not simply a single, linear process, but is highly variable and modular, warranting quantitative frameworks for understanding nuances of the transition.

## Abstract (web summary)

It is unclear if a common EMT expression program exists. Here, the authors perform multiplexed single-cell RNA sequencing across 12 EMT time courses and 16 kinase inhibitor screens, and find that EMT transcriptional responses are context specific and EMT is not a single, linear transition.

## Introduction

Epithelial–mesenchymal (E/M) plasticity is ubiquitous within all epithelial tissues and the reversible transition between these two states contributes to a variety of biological processes, including tumor progression^1^. During the epithelial–mesenchymal transition (EMT), epithelial cells lose defining characteristics, such as stable cell–cell junctions, and gain the capacity to migrate and invade through extracellular matrices^1^. While the EMT has been extensively studied, a variety of EMT responses have been reported and no common, EMT-defining gene expression program has been identified^2^. The transition has historically been depicted as a simple conversion between discrete epithelial and mesenchymal states, but reports of individual cells co-expressing epithelial and mesenchymal genes have since introduced the concept of a partial EMT. This hybrid state has been shown to provide optimal stem cell traits to cancer cells^3,4^, allow for collective tumor cell migration and the formation of circulating tumor cell clusters^5–8^, and is associated with metastatic tumors^9^.

Complicating the definition of epithelial, mesenchymal, and hybrid states, most studies have relied on bulk expression measurements of a small subset of marker genes from a static population of cells. Markers of epithelial and mesenchymal states are likely context specific, and thus relying on a small subset of these markers may lead to erroneous conclusions about the relative E/M status of cells. Single-cell RNA sequencing (scRNA-seq) analysis of head and neck squamous cell tumors demonstrated that while E/M plasticity was evident in many tumors, the specific partial EMT gene signature between tumors was variable^9^. Experimental induction of the EMT can also be variable: microarray analysis of three cell lines exposed to a combination of TGFB1 and TNF alpha (TNF) resulted in EMT responses with only 10–30% of differentially expressed genes shared between conditions^10^. And in a single mammary epithelial cell line, TGFB1 treatment and a spontaneous EMT induction model resulted in different EMT response trajectories, with only approximately a 50% overlap in differentially expressed genes^11^. This variability is also not limited to transcriptomic data, and canonical E/M proteins also co-occur inconsistently^12^.

The extent of variability among EMT programs and the regulatory networks that drive them is still unclear given that most evidence spans multiple independent studies, and few have performed controlled comparisons. Here, we provide a thorough comparison of experimentally induced EMTs, spanning multiple cell types, and EMT inducers. We leverage highly multiplexed scRNA-seq to assess context specificity of the EMT and to compare regulatory features of the transition, assessing 103,999 cells from 960 samples, comprising 12 EMT time course experiments and kinase inhibitor screens for each.

## Results

### Multiplexed scRNA-seq enables comparative analysis of the EMT

To assess transcriptional dynamics of the EMT across a variety of contexts, we used MULTI-seq^13^ to generate scRNA-seq data from 12 distinct EMT time course experiments. We assessed four different cancer cell lines capable of undergoing an EMT (A549, lung; DU145, prostate; MCF7, breast; and OVCA420, ovarian) and exposed each to known EMT-inducing factors: TGFB1, EGF, and TNF. These cell lines were chosen because they all have an epithelial morphology in culture, have been shown to undergo an EMT in previous studies^14–20^, and represent four distinct cancer types. The specific inducers were chosen as they all have been previously shown to promote an EMT in different cell lines—including those used in this study in most cases^14–20^—and their binding to each of their cell surface receptors initiates independent signaling pathways. In response to these factors, each cell line exhibited morphological changes, consistent with an EMT (Supplementary Fig. 1a). We note different inducers can promote different morphologies in the same cell line (e.g., DU145 with TGFB1 or TNF), and some changes were modest in comparison to others (e.g., MCF7 cells treated with EGF). Lacking a typical spindle-shaped morphology, however, does not preclude other EMT traits. For example, at higher doses, EGF has been shown to promote an EMT associated with a circular morphology^21^. Ultimately, it is likely that these differences arise from subtleties in the expression programs initiated by each inducer, and particularly the different expression dynamics of various cytoskeletal and extracellular matrix proteins.

**Fig. 1:**
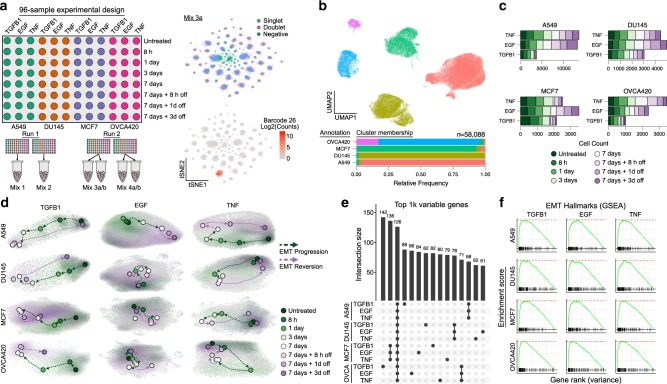
Multiplexed scRNA-seq profiling of 12 EMT time course experiments. **a** Schematic of the 96-well experimental design for the 12 EMT time course experiments (left), and *t*-SNE embeddings of the MULTI-seq barcode counts, demonstrating strong signal for demultiplexing (right). **b** UMAP embedding of aggregated expression data of all data, colored by unsupervised clustering (top), and a graph showing the relative proportion of annotations for each cell line assigned to each cluster after demultiplexing (bottom). **c** Graph showing the number of cells captured for each time course experiment. **d** UMAP embeddings of each of the 12 time course experiments. Grey dots correspond to individual cells, shaded regions represent the related sample density for each time point, and colored dots correspond to the maxima of the density function. **e** UpSet plot showing the intersections of the top 1000 variable genes of each time course experiment. **f** GSEA plots showing the NES for the EMT hallmark genes in the variance-ranked genes for all conditions.

For each of the 12 conditions, samples were collected at five distinct time points from 8 hs to 1 week after treatment, and three additional time points from 8 h to 3 days after the EMT-inducing stimulus had been removed (Fig. 1a). The 3-day withdrawal time point was chosen based on preliminary data, suggesting transcriptional reversion in as few as 3 days. In the aggregated data, expression profiles clustered dominantly by cell line, and after demultiplexing, the majority of cell line annotations (95.8% on average) were restricted to a dominant cluster, demonstrating robust multiplexing (Fig. 1b, Supplementary Fig. 1b). In total, we annotated 58,088 single cells from across 576 samples, comprising six replicates of the 12 time course experiments (Fig. 1c, Supplementary Fig. 1c). Replicates were highly correlated, supporting the consistency of the experimental procedures and processing workflow (Supplementary Fig. 2).

### Transcriptional dynamics of the EMT are context specific

We next assessed the temporal progression of each time course. In each case, time-dependent shifts in cells’ expression profiles were evident, and withdrawal samples showed reversion back toward the untreated state (Fig. 1d). In each cell line, receptors for the three EMT inducers were detectable, explaining these dynamics (Supplementary Fig. 3). While the top 1000 variable genes for each time course showed some expression patterns conserved across cell lines, context-dependent gene sets were dominant (Fig. 1e). Gene set enrichment analysis (GSEA) of variance-ranked genes for each time course did, however, demonstrate enrichment for the MSigDB hallmark EMT gene set in all conditions^22^ (Fig. 1f). This is consistent with the morphological changes we observed, and further supports that these changes are associated with an EMT response. The minimal overlap of top variable genes among conditions suggests that the specific EMT genes involved in the response may vary.

**Fig. 2:**
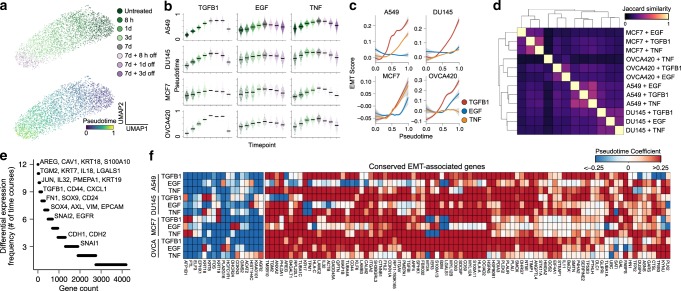
EMT transcriptional responses are largely context specific. **a** UMAP embeddings of A549 cells treated with TGFB1. Each point represents an individual cell, and colors correspond to time point (top) or pseudotime value (bottom). **b** Sina plot showing the distribution of pseudotime values across time points for all 12 time course experiments, with time points colored the same as in **a**. Horizontal black bars represent mean expression values for each group and each point corresponds to a single cell. **c** Smoothed model of the EMT hallmark gene set score throughout the pseudotime. Shaded bands for each line correspond to the standard error for each model. **d** Clustered heatmap of all pairwise Jaccard similarity values for the differentially expressed genes in each condition. **e** Counts of how frequently each gene is differentially expressed among time course experiments. **f** Heatmap showing EMT-associated expression changes associated with a gene set of all genes that are differentially expressed in at least eight time course experiments. The colormap corresponds to the pseudotime beta coefficient of the linear model for each gene.

To specifically compare temporal dynamics of the EMT, we first pseudotemporally ordered the cells from each condition (Fig. 2a, b). In each time course, cells progressively transitioned throughout the full 7 days of EMT induction, and withdrawal of the EMT stimulus led to a near-complete reversion after as few as 3 days (Fig. 2b). We note that it is possible that the cells could have continued to transition following day 7. It will be important for future studies to assess the temporal limits of the EMT response. We then assessed gene expression dynamics throughout the pseudotemporal trajectories. In all cases, transitions were not simply linear processes of two opposing E/M expression programs. Rather, all involved combinations of discrete transcriptional events (Supplementary Fig. 4), suggesting that the EMT may be a multistep process. We found that each condition, with the exception of A549 cells induced with EGF and OVCA420 treated with TNF, was associated with an average increase in the expression of the EMT hallmark gene set^22^, with TGFB1 often producing the most potent effects (Fig. 2c). GSEA revealed, however, that differentially expressed genes from these two conditions, along with all others, were enriched for the hallmark gene set (Supplementary Fig. 5a), but in these two specific conditions, several EMT hallmark genes are repressed, resulting in a net neutral EMT score (Supplementary Fig. 5b).

Surprisingly, responses of individual cell lines to different stimuli were more similar than the responses of different cell lines to the same stimulus, but importantly, all pairwise comparisons show very little overlap in their differentially expressed genes (average Jaccard index of 0.22; Fig. 2d). Of all genes differentially expressed across conditions, the majority changed in as few as one to two conditions, suggesting that the global expression programs associated with the EMT are remarkably context specific (Fig. 2e, Supplementary Data 1). A small subset of canonical EMT genes, including TGFB1, CD44, and FN1, along with less-reported genes, such as TGM2 and PMEPA1, were differentially expressed in most conditions. The majority of the MSigDB hallmark EMT gene set was differentially expressed in only a small number of conditions, with only 49/200 hallmark genes being differentially expressed across the majority of conditions (Supplementary Fig. 6). Extracellular matrix proteins, proteases, and integrins from the hallmark gene set are variably affected across conditions, which could explain the differences in morphological changes observed (Supplementary Fig. 6). This may reflect that the hallmark genes were derived from various founder gene sets that may have been driven by fibroblast expression rather than an EMT (ref. ^23^). Interestingly, however, many canonical EMT genes, including SNAI1, CDH1 (E-cadherin), and CDH2 (N-cadherin) differentially expressed in only a small number of conditions (Fig. 2e).

To identify signatures that may not have been represented in the hallmark gene set, we took all genes that were differentially expressed in at least eight (defined as two-thirds of our conditions as to not be too restrictive) of our experimental conditions, and compiled our own gene set of 86 conserved upregulated genes and 17 downregulated genes (Fig. 2f, Supplementary Data 2). While no gene represents a universal marker of the transition, this list contains those that were most frequently changed. Common epithelial-associated (downregulated) genes included various keratins (*KRT8, KRT18*, and *KRT19*), consistent with morphological changes and the loss of epithelial features. While the conserved mesenchymal-associated (upregulated) genes contain several canonical EMT genes, many are not typically associated with the transition. These upregulated genes, however, do enrich for GO terms associated typical EMT-associated traits, including extracellular matrix disassembly (*p* = 5.0e−4) and organization (*p* = 3.7e−4), cell migration (*p* = 2.0e−3), and negative regulation of apoptosis (*p* = 4.4e−11; Supplementary Fig. 7a). Regulatory regions of the 86 mesenchymal-associated genes are also enriched in binding sites for AP-1, MYC, MEF2, and KLF transcription factors (Supplementary Fig. 7b). These factors have all been implicated in the EMT and could represent conserved regulators of the transition^24–28^. We also confirmed that these 86 mesenchymal-associated genes have variable expression levels among cancer cells from individual human lung tumors and syngeneic mouse tumor models, as well as in scRNA-seq data of healthy epithelium from various mouse tissues (Supplementary Fig. 8a). Further, in each of these data sets, the 86 mesenchymal-associated genes are highly correlated (Supplementary Fig. 8b). Together, this suggests that this expression program is not simply an artifact of culture experiments, but are coexpressed in vivo and may contribute to an E/M heterogeneity program in these tissues.

### The EMT can be coordinated by diverse transcription factor networks

**Fig. 3:**
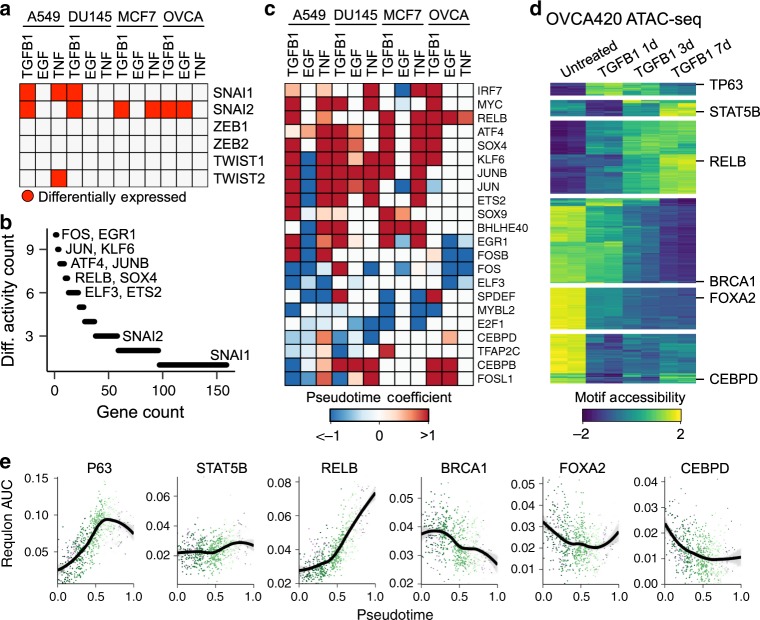
Inferring transcription factor activity throughout the EMT. **a** Plot showing in which time course experiments various canonical EMT transcription factors are differentially expressed. **b** Counts of how frequently various transcription factors and their associated regulons are differentially active among time course experiments. **c** Heatmap showing EMT-associated changes of regulons that are differentially active in at least six time course experiments. The colormap corresponds to the pseudotime beta coefficient of a linear model for each regulon. **d** Differential accessibility of transcription factor motifs from ATAC-seq data of OVCA420 cells treated with TGFB1 for 0, 1, 3, or 7 days. The colormap represents the accessibility *Z*-score for each transcription factor motif. Examples of transcription factors from each cluster are listed. **e** Regulon activity score of the same transcription factors listed in **d** inferred from the OVCA420 TGFB1 time course experiment. Each dot represents a single cell, colored by time point. The black line corresponds to the modeled trend from a generalized additive model. Shaded bands for each line correspond to the standard error for each model.

While many of the most conserved EMT genes are regulated by shared regulatory factors (Supplementary Fig. 7b), these conserved genes only represent a small fraction of differentially expressed genes. We next sought to determine if the remainder of EMT-associated expression dynamics are driven by a common regulatory program that perhaps gives rise to distinct expression patterns due to cells’ epigenetic or mutational profiles, for example. Across the experimental conditions we assessed, most canonical EMT transcription factors—other than SNAI2—were rarely differentially expressed (Fig. 3a). While in some cases (e.g., TWIST1) the transcription factors were not detected, perhaps owing to insufficient sensitivity to lowly expressed genes, canonical EMT transcription factors were often readily detectable, but did not show dynamics throughout the EMT response (Supplementary Fig. 9). We scored each cell for the coexpression of transcription factors and their putative target genes (regulons), and identified those that showed differential activity throughout the EMT. We found that transcription factor activity is also remarkably context specific, with most being restricted to one to two of our time course experiments (Fig. 3b). Several factors were fairly well conserved, however. Consistent with our list of conserved genes, AP-1 (JUN, JUNB), the NFkB-associated RELB, ATF4, SOX4, and KLF6 regulons showed frequent activation, whereas ELF3 and MYBL2 activity often decreased (Fig. 3c). These factors have all been previously implicated in the EMT, but are not typically considered canonical EMT regulators^29–34^. To assess the accuracy of these results, we used ATAC-seq to assess the accessibility of transcription factor motifs throughout the EMT and compared accessibility dynamics to the inferred regulon activity. For the purpose of validation, we chose to assess the OVCA420 TGFB1 time course (Fig. 3d). This was the smallest data set in our scRNA-seq cohort, so we chose to validate the approach on the condition with the lowest power for inferring transcription factor activity. We found that in many cases, motif accessibility throughout the EMT-mirrored regulon activity measured from scRNA-seq data alone (Fig. 3e). This supports that the regulon activity inference provides an accurate representation of the transcription factor activity throughout each of the conditions assessed.

### Kinase inhibitor screens reveal signaling dependencies in a variety of EMT responses

**Fig. 4:**
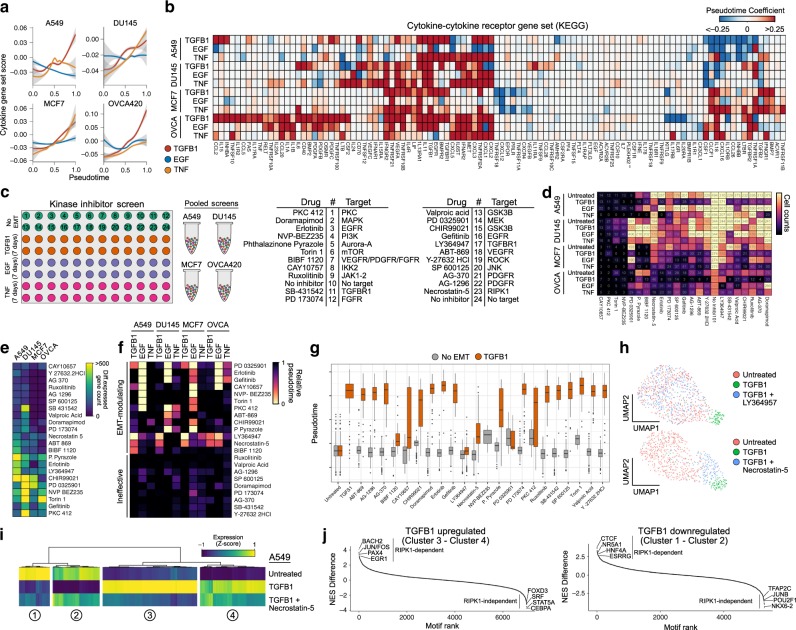
Kinase inhibitor screens identify signaling dependencies of the EMT. **a** Gene set score of the KEGG pathway “cytokine–cytokine receptor interaction” over pseudotime for each time course experiment. Shaded bands for each line correspond to the standard error for each model. **b** Heatmap showing EMT-associated changes of the individual genes of the same gene set as in **a**, only listing those with a significant change in at least one time course experiment. **c** Schematic of the 384-sample experimental design for the kinase inhibitor screen. **d** Heatmap showing the number of cells annotated to each condition after demultiplexing the scRNA-seq data. **e** Summary of the number of genes that are differentially expressed in each cell line exposed to the inhibitors without EMT induction. **f** Average pseudotime values calculated for each condition. **g** Boxplots showing the distribution of pseudotime values for A549 cells treated with the inhibitors alone (grey) or in combination with TGFB1 (orange). The horizontal black line of the boxplot represents the median value, the box spans the 25th and 75th percentiles, and whiskers correspond to 1.5 times the interquartile range. **h** UMAP embeddings of untreated A549 cells with those had been treated with TGFB1 alone or in combination with the TGFBR1 inhibitor LY364947 (top), or the RIPK1 inhibitor necrostatin-5 (bottom). **i** Heatmap showing expression (*Z*-score) of genes differentially expressed in A549 cells by TGFB1 in untreated A549 cells, as well as those treated with TGFB1 alone or in combination with necrostatin-5. **j** Difference in normalized enrichment scores for transcription factor targets in genes that are successfully inhibited by necrostatin-5 compared to those that are not. Positive values correspond to regulons with that are enriched in necrostatin-5-inhibited genes, whereas negative values represent those are not affected by necrostatin-5.

Paracrine signaling is another regulatory feature likely to coordinate the EMT across a population of cells^35–38^. In fact, we found that the expression of secreted factors spanning a variety of signaling pathways broadly increased in each of our 12 time courses (Fig. 4a, b). Given this, we next established an experimental design to mechanistically assess the dependence of the EMT on multiple signaling pathways and compare these dependencies across contexts. We curated a selection of 22 small molecule inhibitors targeting a variety of kinases and treated cell lines alone for 7 days, or in combination with one of the three EMT inducers previously used (Fig. 4c). Leveraging MULTI-seq to multiplex samples, we ultimately generated scRNA-seq profiles for 45,911 cells across the 384 distinct conditions.

From retrieved cell counts alone, drop-out patterns from cell line-dependent and -independent cytotoxic/cytostatic effects can be observed (Fig. 4d, e). To assess the impact of these inhibitors on EMT progression, however, we calculated pseudotime values for the inhibited cells using the models built from corresponding time course experiments of the same cell line and EMT inducer (Fig. 4f). From this, we could identify inhibitors that reduced cells’ pseudotime values at 7 days compared to uninhibited controls, therefore dampening the EMT response. LY364947 (TGFBR1 inhibitor), for example, abrogated TGFB1-induced EMTs (Fig. 4f–h), and erlotinib and gefitinib (EGFR inhibitors) consistently blocked the effects of EGF (Fig. 4f).

The effects of these inhibitors, however, weren’t limited to blocking the direct signaling of the EMT-inducing factor. For example, TGFBR1 inhibition partially blocked EMT progression in a variety of conditions, including EGF-treated A549 and OVCA420 cells, and TNF-treated A549 and MCF7 cells (Fig. 4f). This suggests that the activation of paracrine TGFB1 signaling may be critical for EMT progression, following a variety of initial stimuli, supporting previous work showing the dependence of the EMT on transcription-factor-activated TGFB1 autocrine loops^39–41^.

Effects of direct EGFR inhibition with erlotinib and gefitinib were largely restricted to EGF-treated EMT responses, but inhibition of its downstream kinase MEK (with PD 0325901) hindered the EMT response in TGFB1-treated A549 and MCF7 cells. Non-canonical TGFBR1 signaling through MEK/ERK has been previously reported^42,43^, and two recent studies have proposed a MEK-dependent regulatory checkpoint in the EMT (refs. ^11,44^). While our data for TGFB1-treated A549 and MCF7 cells are in agreement with these findings, it also demonstrates that this checkpoint is not universal, even among other TGFB1-induced EMT responses, as DU145 and OVCA420 cells are not susceptible to MEK inhibition (Fig. 4f).

Inhibition of RIPK1—a kinase involved in activating NFkB and necroptosis pathways—with necrostatin-5 (Nec-5) blocked EMT progression in all of the same conditions as TGFBR1 inhibition. Nec-5-treated cells, however, consistently had higher pseudotime values than TGFBR1-inhibited cells, suggesting a partial EMT response (Fig. 4f, g). To determine if the partial response corresponds to reduced magnitude of gene expression changes, or a selective inhibition of a subset of genes, we assessed expression levels of all genes differentially expressed following TGFB1 treatment. In each case, RIPK1 inhibition only abrogated a subset of TGFB1-induced expression changes, producing a partial EMT response (Fig. 4h, i). Importantly, we note that this partial response with RIPK1 inhibition is not due to a temporal block in EMT progression (i.e., preventing late EMT dynamics), as inhibition does not exclusively prevent late response genes (Supplementary Fig. 10). This suggests that the EMT involves multiple independent regulatory modules that can be perturbed without impacting others.

To our knowledge, no direct cross-talk between the TGFB1 signaling and RIPK1 has been documented, but loss of RIPK1 has been previously associated with an enhanced epithelial phenotype, reduced ERK1/2 phosphorylation, and reduced transcriptional activity of the AP-1 complex^45,46^. To determine if RIPK1 inhibition prevents the activation of AP-1 targets in our EMT models, we assessed the enrichment of transcription factor binding motifs in the promoters of genes that failed to change throughout the EMT in RIPK1-inhibited cells. We found that the AP-1 binding site (JUN/FOS, BACH2 motifs) was the most enriched in promoters of genes that failed to become upregulated in Nec-5-treated cells in response to TGFB1 (Fig. 4j). Other enriched motifs include EGR1 and PAX4 binding sites. Both AP-1 and EGR1 can be activated through ERK1/2 signaling, providing a possible mechanistic link between RIPK1 and these transcriptional changes^46,47^. As ERK1/2 is a downstream effector of MEK, this may also explain the previously proposed MEK checkpoint of the EMT (refs. ^11,44^). While it is still unclear how RIPK1 becomes activated, this regulatory axis is conserved in every condition we assessed that is also dependent on TGFB1 signaling (based on similarity to TGFBR1 inhibition), and may represent a common, though not universal, regulatory network of the EMT.

## Discussion

Here, we have demonstrated that the EMT is a complex cellular process, driven by independent regulatory networks that ultimately give rise to incredible context specificity. Given these findings, we argue that the common paradigm of cells simply undergoing a linear transition between well-defined epithelial and mesenchymal programs is an oversimplification that can lead to erroneous conclusions. Given the variety of EMT responses that can be elicited—each with remarkable dissimilarity—a single mesenchymal gene expression program simply does not exist. The variety of possible responses also makes the full EMT indefinable, as the combination of all is likely to never occur. For the same reason, the number of possible partial EMT states is likely inumerable. This partial state has historically been defined as cells co-expressing both epithelial and mesenchymal markers, but studies have often relied on a small number of canonical markers to make this designation, and we have shown that most markers are inconsistently involved in the transition. This does not discount the likely importance of gradation along some axis of epithelial and mesenchymal phenotypes, but a more comprehensive definition of intermediate and polar states is required.

In this study, we have begun to take steps toward understanding the complexity of E/M plasticity. As single-cell technologies are becoming increasingly scalable, it may soon be possible to learn the complete manifold of all possible states related to E/M plasticity for a given cell type. Unique environment and developmental history will likely mean that this manifold will vary for each cell type, but it may be possible to learn models for their prediction or alignment across settings. It will also be critical to understand how positions along the manifold are associated with phenotypic traits, and how cell perturbations promote dynamics within it. With a comprehensive model of the E/M plasticity, we will gain a quantitative understanding of nuanced cellular heterogeneity, improving our understanding of development, tissue homeostasis, and disease progression. This information will also help inform new strategies to therapeutically modulate cellular phenotypes in disease.

## Methods

### Cell culture

A549, DU145, and MCF7 cells were obtained from ATCC (CCL-185, HTB-81, and HTB-22, respectively). OVCA420 cells were kindly provided by Dr. Gordon Mills. All cells were cultured in Dulbecco’s Modified Eagle Medium with 4.5 g/L glucose, L-glutamine, and sodium pyruvate (Corning, 10-013-CV), supplemented with 10% of fetal bovine serum and cultured at 37 °C with 5% CO_2_.

### EMT time course experiments

For each cell line, 10,000 cells were plated into each well of a 96-well plate according to the schematic in Fig. 1a. The addition of TGFB1, EGF, and TNF were scheduled such that all time points completed at the same time for collection. Cells were treated with 10 ng/mL TGFB1 (R&D Systems, #240-B-010), 30 ng/mL EGF (Invitrogen, #PHG0311), or 10 ng/mL TNF (Invitrogen, #PHC3015). Media was changed and fresh TGFB1, EGF, or TNF were added every 2 days to ensure relatively constant concentrations of these factors. To avoid over-confluence throughout the experiments, cells were passaged as required, but not within the last 2 days of the time course to avoid artifacts at the time of collection. After the scheduled treatments, cells were immediately processed for scRNA-seq multiplexing.

The time course experiments were performed twice independently. Each time, the two time course replicates were performed in parallel, and on the second time through the experiment, two 10x libraries were generated for each plate replicate. Samples from the first replicate are labeled “Mix1” and “Mix2”, corresponding to the two plates running in parallel. Samples from the second replicate are labeled “Mix3a/b” and “Mix4a/b”.

### Kinase inhibitor screen

For each cell line, 10,000 cells were plated into four 96-well plates according to the schematic in Fig. 4c. Cells were simultaneously treated with small molecule kinase inhibitors (listed in Fig. 4c) and either 10 ng/mL TGFB1, 30 ng/mL EGF, or 10 ng/mL of TNF. No-inhibitor and No-EMT-inducer controls were also included for all conditions. All inhibitors were used at a final concentration of 1 µM (Cayman Chemical Kinase Screening Library, Item No. 10505, Batch No. 0537554). EMT inducers and kinase inhibitors were refreshed daily after replacing the culture media. After 7 days of treatment, all samples were immediately processed for scRNA-seq multiplexing.

### Multiplexing individual samples for scRNA-seq

Multiplexing was performed according to the MULTI-seq protocol^13^, and reagents were kindly provided by Dr. Zev Gartner. Briefly, culture media was removed and each well was washed with 1× Dulbecco’s phosphate-buffered saline (PBS; Corning, #21-031-CV). Next, a lipid-modified DNA oligonucleotide and a unique sample barcode oligonucleotide were added at 200 nM to 0.05% trypsin with 0.53 mM EDTA. This was added to each sample to be multiplexed, with each sample receiving a different sample barcode. Cells were incubated with this trypsin mixture for 5 min at 37 °C, and plates were gently mixed periodically. After 5 min, a common lipid-modified co-anchor was added to each well at 200 nM to stabilize the membrane residence of the barcodes. Cells were incubated for an additional 5 min at 37 °C with periodic mixing. After this labeling time, all cells were in suspension, lifted from the plate. The trypsin was then neutralized with cultured media, and the cells were mixed by pipetting to ensure a single-cell suspension. Samples were then transferred to V-bottom 96-well plates, and pelleted at 400 × *g* for 5 min. Barcode-containing media was removed, and the cells were then washed with PBS + 1% bovine serum albumin (BSA). Washes were performed twice, and after the final wash, cells were resuspended in PBS + 1% BSA, pooled together, repelleted, and resuspended in PBS + 1% BSA. Viability and cell counts were then performed, before preparation of the scRNA-seq libraries.

### scRNA-seq library preparation and sequencing

Single-cell suspensions were processed using the 10× Genomics Single Cell 3′ RNA-seq kit (v2 for time course experiments, v3 for kinase inhibition). Gene expression libraries were prepared according to the manufacturer’s protocol. MULTI-seq barcode libraries were retrieved from the samples and libraries were prepared independently according to the MULTI-seq library preparation protocol^13^. Briefly, barcode libraries are separated from the cDNA libraries during the first round of size selection in the 10× Genomics library preparation protocol and PCR-amplified prior to sequencing^13^. Final libraries were sequenced on a NextSeq500 (Illumina). Expression libraries were sequenced so that time course libraries reached an approximate depth of 40,000–50,000 reads per cell (for the v2 scRNA-seq kit), and 20,000–25,000 reads per cell for the kinase inhibitor experiment (v3 scRNA-seq kit). For the time course data, we detected a median of 3649 genes and 17,330 UMIs per cell, and for the kinase inhibitor screens, we detected a median of 2360 genes and 7634 UMIs.

### Processing of raw sequencing reads

Raw sequencing reads from the gene expression libraries were processed using CellRanger v2.2.0 for the time course data, and v3.0.2 for the kinase inhibitor data. The GRCh38 build of the human genome was used for both. Except for explicitly setting --expect-cells = 25,000, default parameters were used for all samples. MULTI-seq barcode libraries were simply trimmed to 26 bp (v2 kit) or 28 bp (v3 kit) using Trimmomatic^48^ (v0.36) prior to demultiplexing.

### Demultiplexing expression data with MULTI-seq barcode libraries

Demultiplexing was performed using the deMULTIplex R package (v1.0.2) (https://github.com/chris-mcginnis-ucsf/MULTI-seq). The key concepts for demultiplexing are described in McGinnis et al.^13^. Briefly, the tool takes the barcode sequencing reads and counts the number of times each of the 96 barcodes appears for each cell. Then, for each barcode, it assesses the distribution of counts in cells and determines an optimal quantile threshold to deem a cell positive for a given barcode. Cells positive for more than one barcode are classified as doublets and are removed. Only cells positive for a single barcode are retained for downstream analysis. As each barcode corresponds to a specific sample in the experiment, the sample annotations can then be added to all cells in the data set.

### Data quality control and processing

Quality control was first performed independently on each 10× Genomic library, and all main processing steps were performed with Seurat v3.0.2 (ref. ^49^). Expression matrices for each sample were loaded into R as seurat objects, only retaining cells with >200 genes detected. Cells with a high percentage of mitochondrial gene expression were also removed. We then subsetted the data, making independent seurat objects for each time course or kinase inhibition experiment (i.e., for all independent cell line and EMT inducer combinations). Each condition was then processed independently with a standard workflow. We first removed genes detected in <1% of the cells for the given experiment. The expression values were then normalized with standard library size scaling and log transformation. The top 3000 variable genes were detected using the variance-stabilizing transformation (vst) selection method in Seurat. Expression values were scaled and the following technical factors were regressed out: percentage of mitochondrial reads, number of RNA molecules detected, cycle cycle scores, and for the time course data, batch was also included. For initial exploration, PCA was run on the variable genes, but all UMAP embeddings included in figures are based on PCA run on genes used for pseudotemporal ordering of cells. UMAP embeddings were calculated from the first 30 principal components.

### Pseudotemporal ordering of cells

Pseudotime models for each time course experiment were built using the R package psupertime v0.2.1 (ref. ^50^) on the top 3000 variable genes from each condition. Psupertime is based on ordinal logistic regression, taking scRNA-seq data with sequential labels and identifying a linear combination of genes that places the cells in the specified label order. To build the pseudotime model for each time course, we first omitted the treatment withdrawal samples. Because psupertime is based on regression; however, pseudotime values for new data can be calculated by simply performing matrix multiplication between the coefficient matrix of the pseudotime model and the expression matrix of the new data. We used this approach to calculate pseudotime values for both the treatment withdrawal samples of the time course experiment. We also used the time course models to calculate pseudotime values for the respective kinase inhibition experiments. As the range of pseudotime values can vary between conditions, we simply rescaled the values from 0 to 1 in cases where multiple models were compared in the same figure.

### Differential expression analysis

For time course experiments, expression dynamics of each gene, or transcription factor regulon score, as a function of pseudotime was modeled using the generalized additive model function provided by the R package mgcv with the model exp ~ s(pseudotime, *k* = 4) + batch, with the smoothing parameter estimation method set to restricted maximum likelihood (method = “REML”). The number of basis functions (*k*) was chosen such that the residuals were randomly distributed. *P*-values associated with the smoothed pseudotime function for each gene were adjusted using the p.adjust() function in R with the Benjamini–Hochberg method. As many genes may significantly vary throughout pseudotime but have low effect sizes, we only evaluated significant genes (adjusted *p* < 0.05) that are also within the top 2000 variable genes of each time course experiment. While others may be biologically relevant, their signal in the data is often too low to assess reliably.

When assessing transcription factor activity (Fig. 3) and cytokine production (Fig. 4), we were more generally interested in assessing the directionality of change over pseudotime, so in these cases, we used the same approach, but removed the smoothing function from the model. This allowed us to report the single coefficient associated with the pseudotime covariate, representing whether activity generally increased or decreased throughout the transition.

For the kinase inhibition experiment, we assessed the number of differentially expressed genes in cell lines treated with a kinase inhibitor, but no EMT inducer. For this, we still used the gam() function provided by the mgcv package with the model exp ~ inhibitor, setting the no-inhibitor controls as the intercept. We then quantified the number of genes with an adjusted *p* < 0.05.

### Calculating smoothed expression trends

To calculate smoothed expression trends over pseudotime, we used models used for differential expression, but calculated the fit values for 200 evenly spaced pseudotime values ranging between the minimum and maximum pseudotime values.

### Gene set enrichment analysis

GSEA was performed using the R package fgsea^51^. Input genes were ranked either by their variance values after the vst, computed by Seurat’s FindVariableFeatures() function, or by adjusted *p*-value from the differential expression analysis. Reference gene sets were collected from the Molecular Signatures Database (MSigDB) v6.2.

### Gene set scoring

Gene set scoring of the EMT hallmark gene set and the KEGG pathway “cytokine–cytokine receptor interaction” was performed using the AddModuleScore() function provided by the Seurat package. Default parameters were used.

### Transcription factor regulon scoring of single cells

Regulon scores for individual cells were computed using the SCENIC workflow^52^. Log-transformed expression values for each time course experiment were used as input into the command-line interface functions of pySCENIC. First, gene regulatory networks were computed using the grnboost2 method in the grn function. Next, enriched motifs were identified using the ctx function, providing the cisTarget v9 databases of regulatory features 500 bp upstream, 5 kb centered on the TSS, and 10 kb centered on the TSS. Finally, individual cells were scored for motifs using the aucell function.

### Identifying over-represented transcription factor motifs in gene lists

The R package RcisTarget^52^ was used to identify enriched transcription factor motifs associated with gene lists, using the cisTarget v9 transcription factor motif annotations and the hg19-tss-centered-10kb-10species.mc9nr database of motif rankings. To compare enrichment between two gene lists, we calculated the difference in normalized enrichment scores (NES) for motifs between the two lists and ranked motifs to identify uniquely enriched motifs.

### ATAC-seq sample preparation and analysis

ATAC-seq samples were prepared from OVCA420 cells treated with 10 ng/mL of TGFB1 for 0, 1, 3, or 7 days, and the experiment was performed independently twice. Sample preparation was performed as described by Buenrostro et al.^53^. Briefly, nuclei were extracted from 50,000 cells per sample and chromatin was tagmented using the TDE1 transposase provided in the Nextera DNA Library Preparation Kit (Illumina). While the original protocol recommended 2.5 µL of enzyme, we found that optimal tagmentation of these samples required 5 µL of enzyme at 37 °C for 30 min with gentle mixing. Finally, ATAC libraries were amplified and sequenced on a NextSeq500 150-cycle high output run, yielding ~50 M reads per sample.

Raw reads were aligned to the hg38 build of the human genome using Bowtie2 (ref. ^54^) and peaks were called using MACS2 (ref. ^55^) with the following parameters: -q 0.01 --nomodel --shift -100 --extsize 200 -B --SPMR --broad. Differential motif accessibility was calculated using the R package chromVAR (ref. ^56^). Briefly, the summits of peaks from all samples were merged, and expanded to a 250 bp window, centered on the summit. Motifs from the human_pwms_v2 list included with the package were mapped to the peaks using the matchMotifs() function and then deviations across samples were computed. Significant deviations in motif accessibility were identified using the differentialDeviations() function.

### Reporting summary

Further information on research design is available in the Nature Research Reporting Summary linked to this article.

## Supplementary information

Supplementary Information

Peer Review File

Description of Additional Supplementary Files

Supplementary Data 1

Supplementary Data 2

Reporting Summary

## Data Availability

Raw sequencing files and processed UMI count matrices have been deposited in the NCBI Gene Expression Omnibus under the accession GSE147405. Lung tumour scRNA-seq data previously published by Lambretchs et al.^57^ is available at the ArrayExpress accessions E-MTAB-6653 and E-MTAB-6149. scRNA-seq data from syngeneic mouse tumours by Kumar et al.^58^ is available at the GEO accession GSE121861. Epithelial cell scRNA-seq data from the Tabula Muris Consortium^59^ are available through Figshare (https://doi.org/10.6084/m9.figshare.5968960.v2).

## Code availability

All code used to process data and generate figures is available on a public Github repository at https://github.com/dpcook/emt_dynamics.
