# Supplementary material for: Context specificity of the EMT transcriptional response
Source: Nat Commun. 2020 May 1;11:2142. doi: 10.1038/s41467-020-16066-2 (PMC7195456; doi:10.1038/s41467-020-16066-2)
Supplement: Supplementary file 1 — Supplementary Information [file 41467_2020_16066_MOESM1_ESM.pdf]

Supplementary Information

Context specificity of the EMT transcriptional response

Cook & Vanderhyden

Supplementary Figures

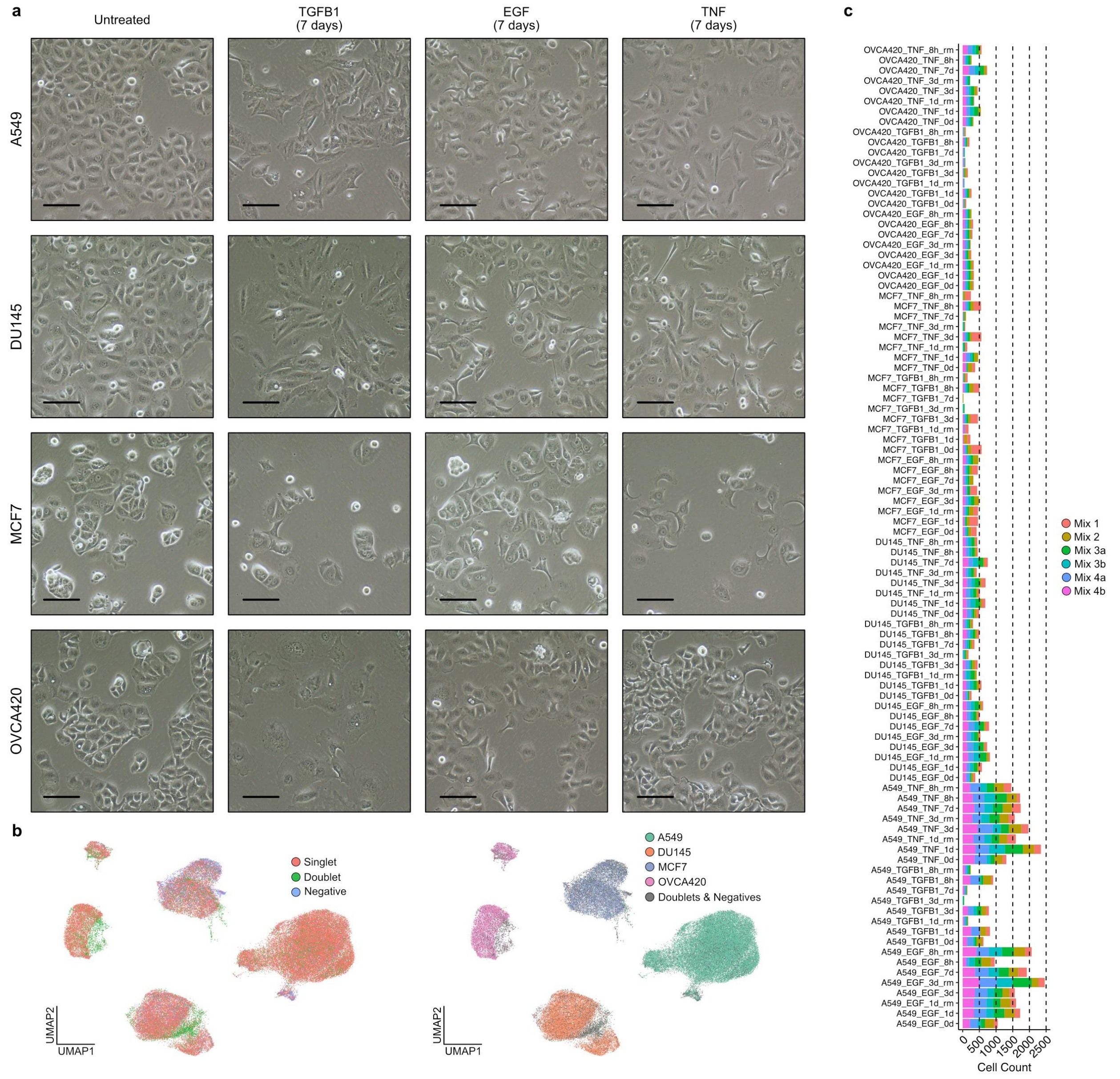

Supplementary Figure 1. Experimental conditions are associated with EMT-associated morphological changes. a, Phase contrast images (20x) of A549, DU145, MCF7, and OVCA420 cells treated with TGFB1, EGF, or TNF. Displayed images are representative fields of all replicates. Scale bar = 100µm**. b,** UMAP embeddings of scRNA-seq data from multiplexed EMT time course experiments. Cells are coloured by their annotation after demultiplexing. **c,** Cell counts for each of the 96 samples of the time course experiments after demultiplexing.

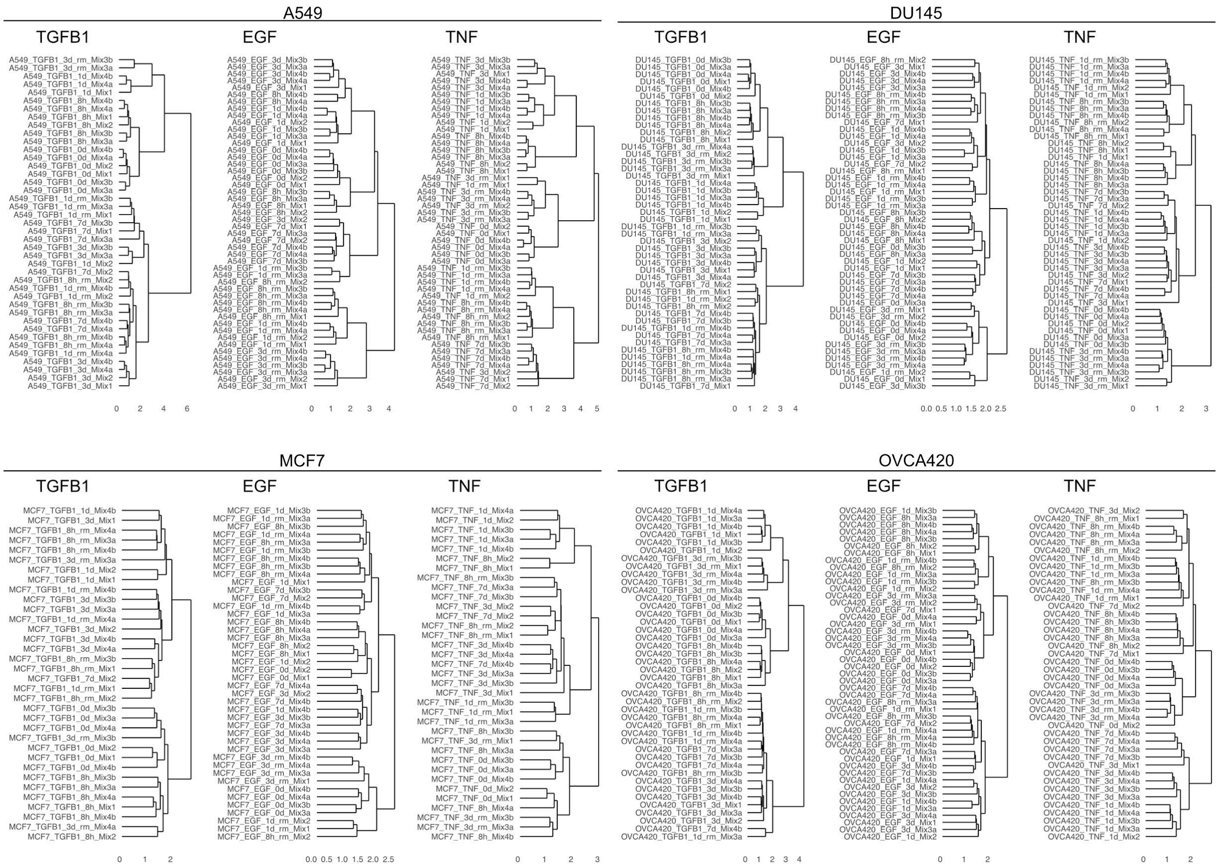

Supplementary Figure 2. Reproducibility of experimental conditions across replicates. Hierarchical clustering of pairwise correlations (Spearman) from scaled expression values of the top 2000 variable genes of each dataset. Scaled expression data includes a simple linear batch correction.

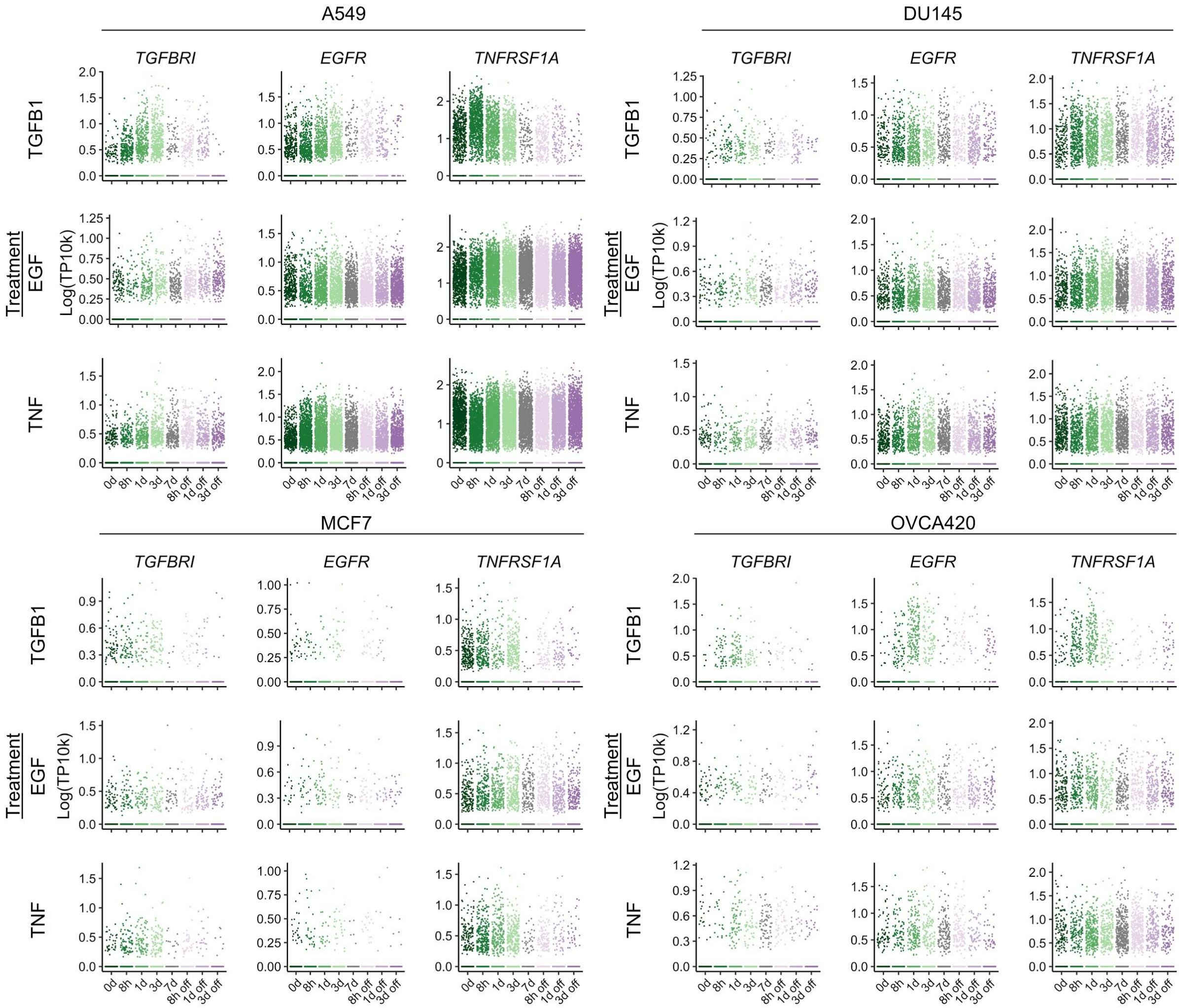

Supplementary Figure 3. Receptors for EMT ligands are expressed in each cell line. Plots showing the expression levels of *TGFBR1, EGFR,* and *TNFRSF1A* in each time course experiment. Expression values are log-transformed counts per 10k molecules (TP10k). Each point corresponds to a single cell.

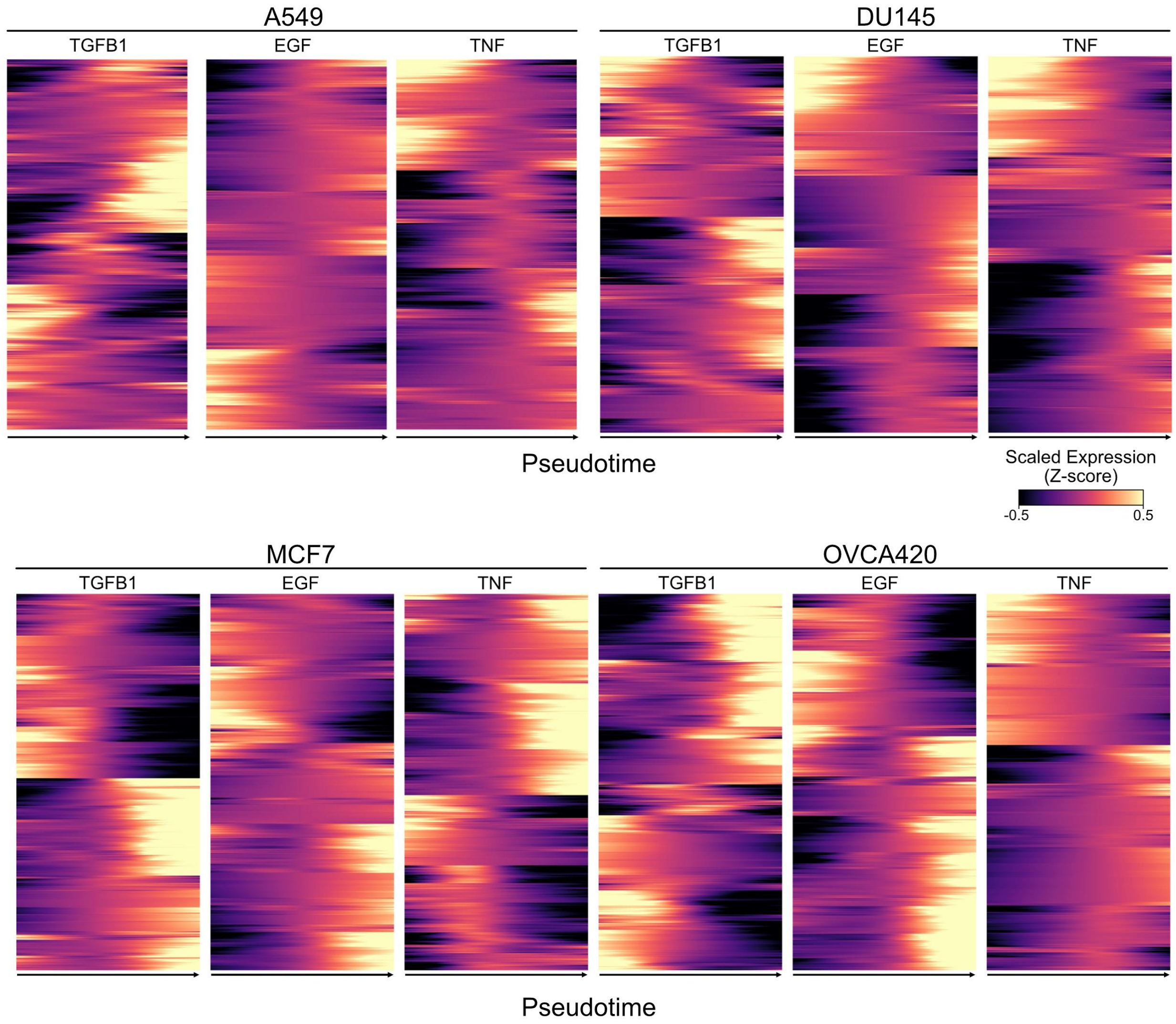

Supplementary Figure 4. The EMT involves multiple waves of transcriptional events. Heatmaps showing expression dynamics of genes differentially expressed in each time course experiment over pseudotime. The x-axis of the heatmaps correspond to EMT progression (pseudotime).

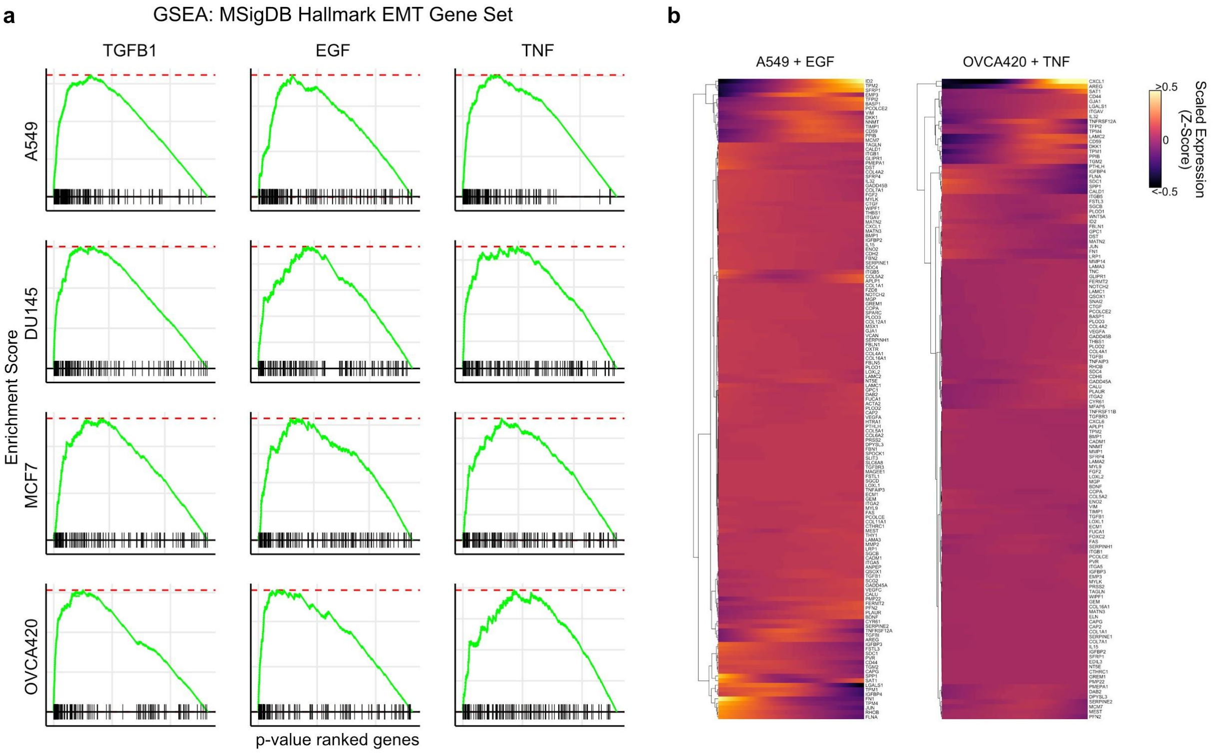

Supplementary Figure 5. EMT-associated variability is present in all conditions. a, GSEA plots showing the enrichment of the MSigDB EMT hallmark gene set in the most differentially expressed genes for each time course. **b,** Expression dynamics of the MSigDB EMT hallmark gene set throughout pseudotime in A549 cells treated with EGF (left) and OVCA420 cells treated with TNF (right).

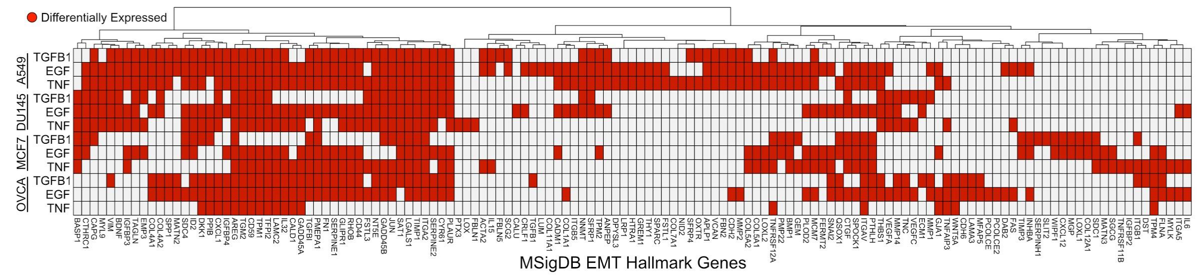

Supplementary Figure 6. Hallmark EMT gene set is variably expressed across conditions. Heatmap showing differential expression (red) of the MSigDB Hallmark EMT gene set across conditions. The plot only includes genes that are differentially expressed in at least one condition.

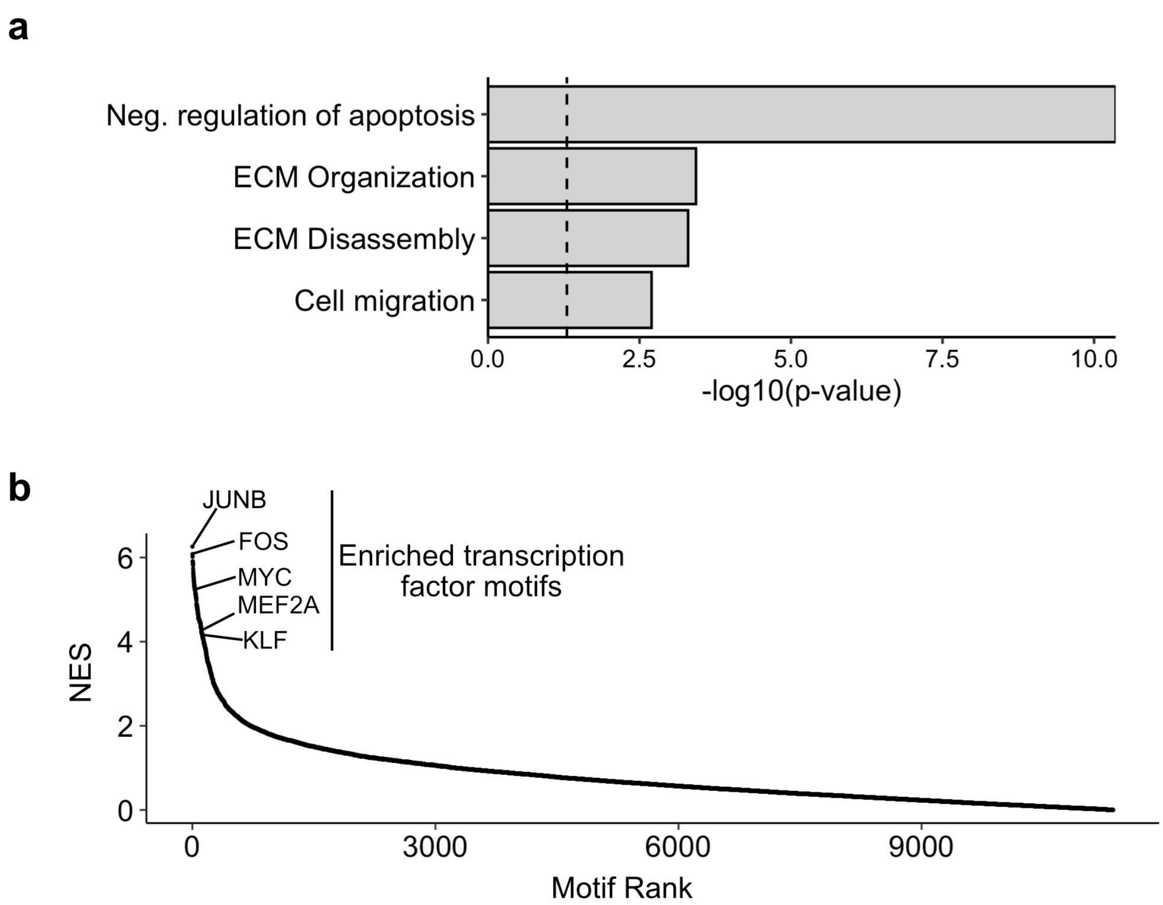

Supplementary Figure 7. Characteristics of conserved EMT genes. a, Select GO Terms associated with the 86 genes commonly upregulated during the EMT. The dashed vertical line corresponds to p=0.05. P-values were calculated from a Fisher's exact test with a Benjamini-Hochberg false discovery rate correction for multiple comparisons. **b,** Normalized enrichment scores (NES) of transcription factor motifs enriched in the regulatory regions of the 86 mesenchymal-associated genes.

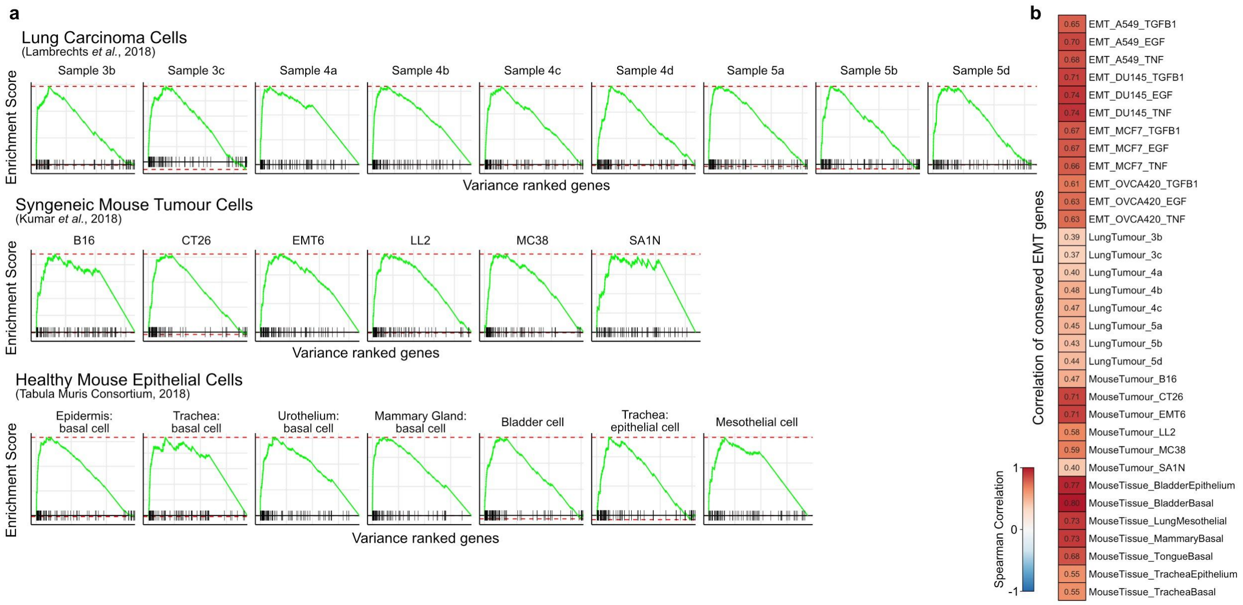

Supplementary Figure 8. Conserved EMT-associated genes contribute to a coordinate heterogeneity program in epithelial tissues and tumours. a, GSEA plots of conserved EMT genes in the most variable genes from a variety of epithelial and carcinoma populations. The conserved EMT gene set corresponds to the 86 genes that are upregulated in at least 8 time course experiments. Human lung carcinoma scRNA-seq data was acquired from Lambrechts *et al.*[57], syngeneic mouse tumour data was acquired from Kumar *et al.*[58], and the healthy mouse epithelium data was acquired from the Tabula Muris Consortium[59]. **b,** The average correlation (Spearman) of the same conserved EMT genes and data sets as in (a).

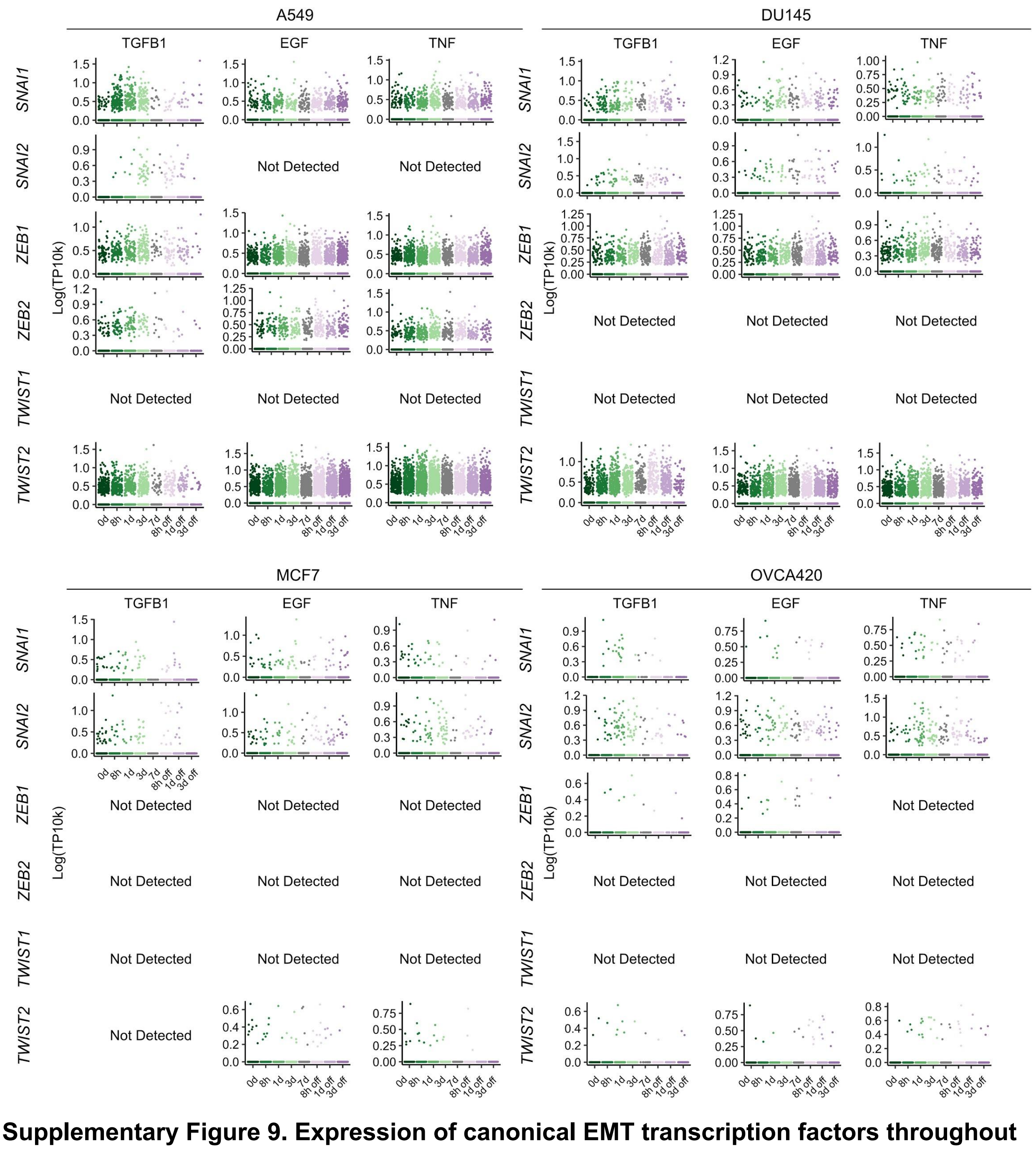

Supplementary Figure 9. Expression of canonical EMT transcription factors throughout EMT responses. Plots showing expression levels of *SNAI1, SNAI2, ZEB1, ZEB2, TWIST1,* and *TWIST2* in each time course experiment. Expression values are represented as log-transformed counts per 10k molecules (TP10k). Each dot corresponds to a single cell.

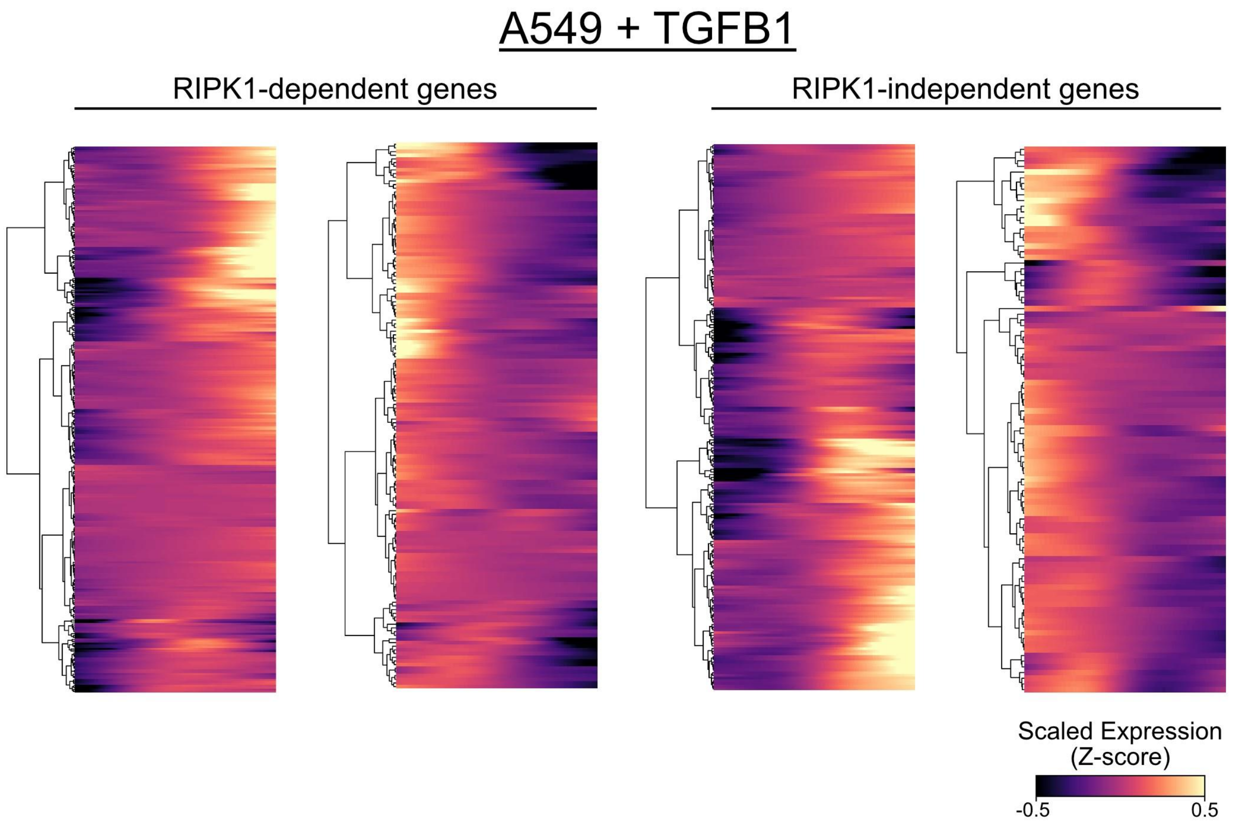

Supplementary Figure 10. Expression dynamics of RIPK1-dependent and -independent genes. Heatmaps of genes whose EMT-associated dynamics during TGFB1 treatment in A549 cells are blocked by RIPK1 inhibition (Nec-5 treatment; left), and those that are not affected by inhibition (right). The x-axis of the heatmaps correspond to EMT progression (pseudotime).
