# Supplementary material for: Context specificity of the EMT transcriptional response
Source: Nat Commun. 2020 May 1;11:2142. doi: 10.1038/s41467-020-16066-2 (PMC7195456; doi:10.1038/s41467-020-16066-2)
Supplement: Supplementary file 3 — Description of Additional Supplementary Files [file 41467_2020_16066_MOESM3_ESM.pdf]

Description of Additional Supplementary Files

File Name: Supplementary Data 1
Description: Tables of differentially expressed genes for each time course experiment.

File Name: Supplementary Data 2
Description: List of conserved EMT genes from time course experiments.
