# Supplementary material for: Context specificity of the EMT transcriptional response
Source: Nat Commun. 2020 May 1;11:2142. doi: 10.1038/s41467-020-16066-2 (PMC7195456; doi:10.1038/s41467-020-16066-2)
Supplement: Supplementary file 6 — Reporting Summary [file 41467_2020_16066_MOESM6_ESM.pdf]

nature research

Corresponding author(s): David P. Cook

Last updated by author(s): Mar 31, 2020

Reporting Summary

Nature Research wishes to improve the reproducibility of the work that we publish. This form provides structure for consistency and transparency in reporting. For further information on Nature Research policies, see Authors & Referees and the Editorial Policy Checklist.

Statistics

For all statistical analyses, confirm that the following items are present in the figure legend, table legend, main text, or Methods section.

n/a	Confirmed	
☐	☒	The exact sample size (*n*) for each experimental group/condition, given as a discrete number and unit of measurement
☐	☒	A statement on whether measurements were taken from distinct samples or whether the same sample was measured repeatedly
☐	☒	The statistical test(s) used AND whether they are one- or two-sided<br>*Only common tests should be described solely by name; describe more complex techniques in the Methods section.*
☐	☒	A description of all covariates tested
☐	☒	A description of any assumptions or corrections, such as tests of normality and adjustment for multiple comparisons
☐	☒	A full description of the statistical parameters including central tendency (e.g. means) or other basic estimates (e.g. regression coefficient) AND variation (e.g. standard deviation) or associated estimates of uncertainty (e.g. confidence intervals)
☐	☐	For null hypothesis testing, the test statistic (e.g. *F*, *t*, *r*) with confidence intervals, effect sizes, degrees of freedom and *P* value noted<br>*Give P values as exact values whenever suitable.*
☒	☐	For Bayesian analysis, information on the choice of priors and Markov chain Monte Carlo settings
☒	☐	For hierarchical and complex designs, identification of the appropriate level for tests and full reporting of outcomes
☐	☒	Estimates of effect sizes (e.g. Cohen's *d*, Pearson's *r*), indicating how they were calculated

Our web collection on statistics for biologists contains articles on many of the points above.

Software and code

Policy information about availability of computer code

Data collection	No software was used for data collection
Data analysis	Code to reproduce all findings for this study is available at https://www.github.com/dpcook/emt_dynamics<br>Raw sequencing data was processed using CellRanger v2.2.0 (Time course data) and v3.0.2 (kinase screen data)<br>scRNA-seq analysis was primarily performed using the R package "Seurat" v.3.0.2<br>R packages used include:<br>psupertime v0.2.1<br>deMULTIplex v1.0.2<br>fgsea v1.12.0<br>mgcv v1.8-31<br>RcisTarget v1.6.0<br>chromVAR v1.8.0<br><br>Command line tools include:<br>Trimmomatic v0.36<br>pySCENIC v0.9.18<br>Bowtie2 v2.3.2<br>MACS2 v2.2.5

For manuscripts utilizing custom algorithms or software that are central to the research but not yet described in published literature, software must be made available to editors/reviewers. We strongly encourage code deposition in a community repository (e.g. GitHub). See the Nature Research guidelines for submitting code & software for further information.

Data

Policy information about availability of data

All manuscripts must include a data availability statement. This statement should provide the following information, where applicable:

- Accession codes, unique identifiers, or web links for publicly available datasets
- A list of figures that have associated raw data
- A description of any restrictions on data availability

Raw sequencing files and processed UMI count matrices have been deposited in the NCBI Gene Expression Omnibus under the accession GSE147405

Field-specific reporting

Please select the one below that is the best fit for your research. If you are not sure, read the appropriate sections before making your selection.

☒ Life sciences ☐ Behavioural & social sciences ☐ Ecological, evolutionary & environmental sciences

For a reference copy of the document with all sections, see nature.com/documents/nr-reporting-summary-flat.pdf

Life sciences study design

All studies must disclose on these points even when the disclosure is negative.

Sample size	Strict sample sizes were not selected a priori. Biological interpretation is limited by statistical significance. As cell lines are a relatively homogeneous population, we reasoned that as few as a couple dozen cells per sample would be sufficient to capture a representative gene expression profile of the population. We designed the study to capture several hundred cells per sample, on average. This targeted yield was based on studies that assessed the number of cells required for gene level and signature level effects on transcription (Dixit et al., Cell, 2016), as well as the upper limit of cells that can be captured from each sample lane on the 10x Genomics Chromium Controller when samples are multiplexed and superloaded (Stoeckius et al., Genome Biology, 2018).
Data exclusions	The use of barcode-based multiplexing allowed us to identify intersample cell doublets in our data (ie. positive for two sample barcodes). These were removed from all downstream analysis. These exclusions were established prior to the study and were made because the goal of single-cell analysis is to study gene expression at single-cell resolution. Doublets are technical artifacts and confound this analysis.
Replication	Replicate strategies are clearly stated in the manuscript. For time course data, a total of six replicates were performed: two independent runs on different days, with each run including two independent replicate plates from different cell stocks, and for the second run, each plate was split into two separate pools (eg. Mix3a/b, Mix4a/b) of cells prior to loading into different sample lanes on the 10x Genomics Chromium Controller. The kinase inhibitor screens were not replicated due to the size of the experiment, but internal controls were included in the design to ensure validity of the experiment. All attempts at replication were successful.
Randomization	Randomization was largely not applicable for this study given that our design had controls and experimental treatment samples for each cell line. Replicate experiments were used to assess batch effects, and between replicates, samples were labeled with different multiplexing barcodes to ensure no barcode-specific effects. No such effects were documented.
Blinding	Blinding was not relevant to this study as ground-truth sample identification is required to model gene expression as a function of the experimental treatment.

Reporting for specific materials, systems and methods

We require information from authors about some types of materials, experimental systems and methods used in many studies. Here, indicate whether each material, system or method listed is relevant to your study. If you are not sure if a list item applies to your research, read the appropriate section before selecting a response.

Materials & experimental systems

n/a	Involved in the study	
☒	☐	Antibodies
☐	☒	Eukaryotic cell lines
☒	☐	Palaeontology
☒	☐	Animals and other organisms
☒	☐	Human research participants
☒	☐	Clinical data

Methods

n/a	Involved in the study	
☒	☐	ChIP-seq
☒	☐	Flow cytometry
☒	☐	MRI-based neuroimaging

Eukaryotic cell lines

Policy information about cell lines

Cell line source(s)	A549, DU145, and MCF7 cells were obtained from ATCC (CCL-185, HTB-81, and HTB-22, respectively). OVCA420 cells were kindly provided by Dr. Gordon Mills (sourced originally from ascites of an ovarian cancer patient by Dr. Robert Knapp)
Authentication	Cell lines were authenticated at their source prior to acquisition, but not further authentication was performed. Early stocks of each line were used for this study to minimize risk of cell line contamination
Mycoplasma contamination	All cell line stocks were tested for mycoplasma contamination prior to freezing. All lines were negative for contamination.
Commonly misidentified lines (See ICLAC register)	None. All cell lines were chosen based on their use in prior EMT literature. Early cell stocks following acquisition were used to minimize risk of cell line contamination.
