# Supplementary material for: Context specificity of the EMT transcriptional response
Source: Nat Commun. 2020 May 1;11:2142. doi: 10.1038/s41467-020-16066-2 (PMC7195456; doi:10.1038/s41467-020-16066-2)
Supplement: Supplementary file 2 — Peer Review File [file 41467_2020_16066_MOESM2_ESM.pdf]

Reviewers' comments:

Reviewer #1 (Remarks to the Author): Expertise in ATAC-seq and EMT

This article entitled « Context specificity of the EMT transcriptional response" is proposed by David P. Cook & Barbara C. Vanderhyden and is interested in the identification of robust/universal transcriptional signature of EMT. Several articles already reported EMT-related genes lists and others demonstrated that no doubt there is not a single EMT program but several EMT, and cells which are more or less engaged in mesenchymal phenotypes. However, the method used in this manuscript is interesting in the way that the authors systematically analyzed transcriptomes using scRNA-seq in 4 different cell models +/- 3 EMT inducers in a kinetic. They also analyzed the effect of +/- 16 kinase inhibitors in these transcriptional programs. To do that, they generated about 104 000 transcriptomes. Although this attractive strategy and impressive number of data, this article lacks a clear message and is too preliminary for be considered in Nature communications.

Specific Comments:

The choice of the different cell lines and EMT-inducers is not clear. A549 cells are frequently used in the literature for studying EMT (since these cells are not fully epithelial cells), but what about the others? Moreover, the paper is constructed on the analysis of the transcriptomes obtained following the exposition to 3 different EMT-inducers. Not clear that all 3 inducers could really induce EMT in the 4 different cell lines. Some specific cell lines are wells described to respond to a specific inducer. Could we consider all the transcriptomes without scoring the EMT status? Pictures presented in Supp Fig1 suggest that many conditions are not associated with EMT (MCF7 with TGF/TNF/EGF, DU145 with EGF, OVCA420 with EGF/TNF). Authors could detect modifications of transcriptomes following treatment but not clear here whether these modifications are obviously related to EMT. Western-blot with E-CADH/N-CADH/VIM status, length of cell measurement… may help to determine which transcriptomes have to be considered. Idem, profiles of UMAP embedding for EGF conditions look like very different. What does it means (no EMT ?...)

Authors chose to take genes that were differentially expressed in at least 8 of their conditions. This cut-off is not explained. The paper is lacking a solid message. The authors proposed a list of 86 genes that they also found in variable cancer cells "as well as healthy epithelium from various mouse tissues". This last sentence is not clear? Not convincing if the genes are found in cancer models and healthy tissues. What is the specificity of these genes? Are these genes associated/correlated with clinical parameters in patients/models (progression free survival, metastasis, migration…)?
May these genes a common regulation (what about transcription factor binding prediction in the promoters? common epigenetic regulation?)

All along the manuscript, it is difficult to understand what is experimentally done without be obliged to constantly refer to Mat & section or tables. (few examples: "EMT time course", "several EMT genes, "variety of effects", "no EMT controls"…) Some details should be added in the main text. Why authors used these 22 inhibitors (does it refers to the 16 molecules written above in the manuscript ?) is not evident to understand.

The name of the target of the molecule under brackets is confusing. Ex: "LY364947 (TGFBR1)" should be replaced by "LY364947 (TGFBR1i)"

The authors showed that inhibition of EGF may partially block induction via TGF. This should be better explained. Could the MAPK pathway be involved in this process since MAPK is in a crosstalk between the non smad pathway (TGFBR) and the EGFR pathway. Molecular experiments are needed to support this observation.

Kinase inhibitors are used concomitantly with EMT inducers. It could be useful to repeat some

experiments with a pre-treatment (e.g. one day before) with the inhibitors to analyze whether inhibition of the target kinases could really block EMT.

The specific choice of the OVCA420 cells for the ATAC-seq experiment is not explained

Some papers previously reporting data also observed in the present paper should be cited.
Gemmil RM et al 2011 Cancer Lett showing that cancer cells are not epithelial or mesenchymal but frequently between these phenotypes.
Peixoto P et al 2019 Cell Death Dis, showing with transcriptomes in different cell models that gene expression was more closely dependent from the cell line than the EMT induction.
Du L et al Cancer Med, analyzing the signature of TGFb-induced EMT
Cieslik M et al Epigenetics Chromatin showing the links between epigenetics and transcriptomes in EMT

Reviewer #2 (Remarks to the Author): Expertise in single-cell seq and EMT

Cook et al. offer a comprehensive single cell transcriptomic analysis of EMT that explores a variety of cancer cell lines exposed to different EMT inducers with a time series experiment that also includes a drug inhibition study. Data is generated through multiplexed single cell RNA sequencing. The resulting dataset is rich and will likely serve as a landmark dataset that will be further explored by many others for years to come.

Using the unique data they generated, the authors make several conclusions about EMT. Notably, they find that EMT is a context-dependent process, with transcriptional dependency on the cell line and EMT-inducing factors tested. Four different cell types were probed (A549, lung; DU145, prostate; MCF7, breast; OVCA420, ovarian) and each was separately treated by one of three different EMT-inducing factors (TGFB1, EGF, and TNF). The authors demonstrate that many of the commonly cited EMT Hallmark genes are not differentially expressed after treatment. The authors produce a new EMT gene list that they claim is most invariant to the conditions tested. Their drug screening analysis on the EMT-induced cells with small molecule inhibitors further demonstrates context-dependent effects.

Overall, the study addresses timely and elusive issues in the EMT/cancer field and provides a valuable new dataset as a resource that can be further mined by the broader community. While the authors present the experiments clearly, the take home points derived from the data analytics are overly broad and not unexpected. Indeed, it is already recognized that EMT is a context-dependent and heterogeneous process. The data generated here may be able to quantify the EMT process in an unprecedented manner but the analysis provided is largely descriptive and leaves several important questions unanswered. Listed below are such questions, together with comments that point to potential limitations of the study.

1) Given the vast number of EMT studies, including recent single-cell analysis studies (scRNAseq specifically), why do the authors not mention those studies and put their work in that current landscape? Some important and highly relevant studies include Pastushenko et al “Identification of the tumour transition states occurring during EMT”, van Dijk et al: “Recovering gene interactions from single-cell data using data diffusion.” There are many others.

2) Given that the data is generated with single cell resolution, there is surprising little to no analysis of cell clusters that may be similar or unique to the various conditions tested. For example, some relevant cell clusters may be the partial EMT states previously reported in the literature. The data that the authors have generated offer the opportunity to explore this possibility and frame the results of this study into a more meaningful context for the broader field.

3) The authors claim that the EMT hallmark gene set contains many genes that are not differentially expressed by any of cancer cell lines treated by various EMT-inducing factors. This finding is not surprising as it has been documented that some of the genes in the EMT Hallmarks are derived from stromal cells (i.e. fibroblasts) in the tumor microenvironment that coordinate the mesenchymal transformation of the epithelial malignant cells through paracrine signaling. These stromal cell types are captured in bulk tumor gene expression studies. Many deconvolution efforts are demonstrating the heterogeneity of cell types contributing to bulk-derived gene expression signatures.

4) The authors provide a new EMT gene list that they claim is most invariant to the conditions tested and more suitable for assessing EMT, despite its context-dependence. However no discussion is provided on the new genes being proposed and why they may be biologically relevant to EMT. This is a limitation of the paper, given that the gene list is a main result of the paper. In addition, it could be instructive to examine if specific genes from their list are altered during the kinase inhibitor treatments. Such a finding could explain the effects of the inhibitors in the context of the authors' main findings.

5) The definition of EMT across the different conditions seems to be “whatever happens after 7 days under the influence of the EMT inducer” regardless of the cell line and the treatment condition. This limitation is not addressed. From the pictures provided in Extended Data Figure 1, EMT-related morphological changes are not as evident in some cell lines under certain conditions. Do all the cell lines express the receptors of the given ligands? Can references be provided as to whether all these ligands induce EMT in these cell lines? For the MET process, it is not clear that under all conditions all cells reverted after 3 days as stated for Figure 2b. Was there a reason a longer withdrawal time-course was not tested?

6) Are there any data showing that the direct targets of all inhibitors used in Figure 4C are present in these cell lines? Protein levels would be preferable even though the paper focused on RNA. Although all effects are assessed relative to EMT “output”, this would shed light on whether some of the results are off target effects. Also, given that the results of Figure 4 are assessed as an EMT dependence on paracrine signaling, it would be helpful to annotate which paracrine signaling is inhibited with each inhibitor. For example, PI3K kinase is involved in many different signaling pathways (not only paracrine), and has been implicated with many of the molecules stated to be inhibited by the drugs tested.

7) Regarding the discussion around the results from RIPK1inhibition, how are EMT and TGFb related to necroptosis? What does blocking cells “half way into EMT” mean? Are the authors referring to a partial EMT state? Are the effects of RIPKI (and some of the other inhibitors) confirmed by EMT-related morphological assessment? Do these drugs inhibit TGFb-induced EMT morphological changes? Are any of these results agreeable with some protein data? EMT can manifest at the level of posttranslational modifications of common markers such as E cadherin, (e.g. Aiello et al. 2018).

Reviewer #3 (Remarks to the Author): Expertise in EMT and drug screening

The manuscript submitted by Cook and Vanderhyden reports that epithelial-mesenchymal transition (EMT) involves various transcript expression profiles among four carcinoma cell lines exposed to three distinct EMT inducers.
The study is based on single-cell sequencing at different time-points and in the presence of kinase inhibitors. ATAC-seq was performed in one cell line. Cells from each well were barcoded to allow demultiplexing pooled cells from each of the 96-well plate experimental designs.
Bioinformatics approaches were applied for quality controls and for establishing the most variable gene list across the different time-points of the EMT kinetics. A pseudo-temporal ordering is

provided for each cell line during EMT progression, spontaneous reversal and after reversal by selected drugs interfering with the EMT inducer signaling pathways. The ATAC data obtained with the OVCA420 carcinoma cell line showed good concordance with the transcription profiles.

Comments

It is not clear why, although the authors consider 12 time-points for the growth kinetics, only 7 time-points are described, including 3 time-points for spontaneous reversal.
Only slightly more than half of the 103,999 cells were analyzed.
TNF is not usually used as EMT inducer. What motivated the authors to use this cytokine and which TNF have they used?
The authors have selected 4 cell lines with very distinct phenotypes and degree of responses to each EMT inducer.
Extended Figure 1a shows that A549 lung adenocarcinoma cells exhibit mixed EMT phenotypes, as shown by multiple studies. This cell line is already engaged in the EMT process at baseline; it responds well to the three inducers used in this study, with no obvious cell death. In contrast MCF7—one of the most epithelial-like cell lines of breast carcinoma—is known to be quite refractory to EMT inducers. Extended Figure 1a suggests that very few cells succeed in engaging into EMT only with TNF. A similar comment can be made for the two other cell lines: DU145 is a rather mesenchymal-like cell line that, again, responds differently to the three inducers with quite distinct morphologies. OVCA420 is epithelial-like cell line that exhibits very distinct responses to the three inducers. Again, one can suspect that TGFb1 induces cell death. The cell counts shown in extended Figure1c reflect this issue.
In Figure1d, EGF treatment led to very modest shifts for all 4 cell lines.
The EMT hallmark (Figure1f) shows a similar profile for all conditions. How can this be explained with these very distinct morphologies?
The EMT score used in this study (Figure 2d) is not significantly increased and, in several cases, does not vary in the pseudo-time analysis.
The EMT signature derived from the four cell lines was then used to analyze two databases for lung cancer and one database for normal mouse tissues (Extended Figure 4). The graphs do not reveal any significantly better results than other signatures. For instance, it well known that basal cells have EMT features, and that most tumors exhibit at a partial EMT phenotype (see for instance Tan et al. EMBO Mol Med, 2014).
The authors used 22 small-molecular compounds with variable specificity to induce EMT reversal in the 4 cell lines in the absence and presence of the EMT inducers. Some of these drugs, as expected, dampened the response to the inducers. Not surprisingly, TGFbetaR inhibitor affected EGFR signaling in A549 cells, since TGFbetaR requires receptor tyrosine kinase signaling for activation in a number of cell lines (Derynck and Budi Sci Signal 2019).
The authors conclude that the expression profiles vary considerably in each condition and that only a subset of well-known EMT genes are revealed in this study. This finding is not surprising due to the choice of these 4 cell lines of different origins with distinct EMT features at baseline and variable response to the inducers. Notably, several EMT expression profiling signatures do not include most of the canonical EMT genes, which are often not highly expressed albeit detected by PCR.
Studies of EMT in development show very distinct signaling systems in gastrulation and in subsequent EMT events in morphogenesis and organogenesis. In cancer, it is also apparent that different tumor types will utilize different signaling pathways to gain intermediate EMT stages. There is also increased evidence for intratumoral heterogeneity for the EMT spectrum in individual cancer cells. The authors should consider these concepts in their Discussion.

Referee #1

This article entitled « Context specificity of the EMT transcriptional response" is proposed by David P. Cook & Barbara C. Vanderhyden and is interested in the identification of robust/universal transcriptional signature of EMT. Several articles already reported EMT-related genes lists and others demonstrated that no doubt there is not a single EMT program but several EMT, and cells which are more or less engaged in mesenchymal phenotypes. However, the method used in this manuscript is interesting in the way that the authors systematically analyzed transcriptomes using scRNA-seq in 4 different cell models +/- 3 EMT inducers in a kinetic. They also analyzed the effect of +/- 16 kinase inhibitors in these transcriptional programs. To do that, they generated about 104 000 transcriptomes. Although this attractive strategy and impressive number of data, this article lacks a clear message and is too preliminary for be considered in Nature communications.

Comment regarding general changes to manuscript text

We would like to thank each of the reviewers for taking time to evaluate our manuscript and for providing thoughtful comments. These comments have inspired a number of additions to the text of the manuscript. While we respond to each specific comment below, the following is an outline of some of the more general changes to each section of the manuscript:

Introduction

We have added much more background and justification for our study. We hope that this does a better job at presenting the key questions we hoped to address.

Results

We have improved the clarity of methodological details, including justification for the conditions we assessed, and relevant references. We have also provided a much more thorough interpretation of our data, particularly regarding our list of conserved EMT genes and the impact of the various kinase inhibitors on EMT progression.

Discussion

This section has been completely rewritten. Interpretations of specific pieces of data are largely contained at relevant points of the results section, so the discussion section is fairly concise, focusing on the broad points that we think are critical to convey.

All modifications are highlighted in the revised manuscript.

Specific Comments:

The choice of the different cell lines and EMT-inducers is not clear. A549 cells are frequently used in the literature for studying EMT (since these cells are not fully epithelial cells), but what about the others?

We have added **lines 68-73** to provide better rationale for the conditions we have chosen. We have also referenced studies that have used these specific combinations of cell lines and inducers. In short, cell lines were chosen because they are four distinct cancer types, each with a fairly epithelial morphology, and previous literature supports their ability to undergo an EMT. The EMT inducers themselves were chosen as they have all been shown to induce an EMT (in many cases, in these specific cell lines), and they activate three different signal transduction pathways, avoiding redundancy.

Moreover, the paper is constructed on the analysis of the transcriptomes obtained following the exposition to 3 different EMT-inducers. Not clear that all 3 inducers could really induce EMT in the 4 different cell lines.

We have added **lines 68-73** to justify the choices of cell lines and EMT inducers and have included references (refs. 14-20) where these specific combinations have been previously studied. Further, we have included data to support that receptors for each of the EMT-inducing ligands are expressed in each of the cell lines (**Extended Data Fig 3**).

Some specific cell lines are wells described to respond to a specific inducer. Could we consider all the transcriptomes without scoring the EMT status? Pictures presented in Supp Fig1 suggest that many conditions are not associated with EMT (MCF7 with TGF/TNF/EGF, DU145 with EGF, OVCA420 with EGF/TNF).
We certainly agree that the morphological changes are diverse across conditions (even within the same cell line), but we do believe that the changes are evident. We have added **lines 76-79** to reference an interesting study that showed that at higher doses, EGF can induce mesenchymal traits along with a "circular" morphology rather than the classic elongation. Considering the transcriptome of each condition, all twelve have EMT-associated genes enriched among their differentially expressed genes (**Extended Data Fig. 5a**). Supporting this further, we have extended the analysis and interpretation of the 86 well-conserved genes from our 12 conditions and show enrichment of GO terms associated with common EMT traits (ECM disassembly/organizing, cell migration, negative regulation of apoptosis; **lines 151-154, Extended Data Fig 7a**). Thus, we believe that all molecular evidence supports that each condition is exhibiting changes consistent with an EMT response.

We have also added **Fig 2d** to show that there is remarkable variability in the responses of even the conditions that look like a canonical EMT (low Jaccard similarity of differentially expressed genes). In fact, the individual cell lines' responses to different inducers are more similar than different cell lines to the same inducer. So, for example, DU145 cells treated with TGFB1 (striking morphological changes) have a more similar response to DU145 cells with EGF (weak morphological changes) than A549 cells treated with TGFB1 (striking changes).

Authors could detect modifications of transcriptomes following treatment but not clear here whether these modifications are obviously related to EMT. Western-blot with E-CADH/N-CADH/VIM status, length of cell measurement… may help to determine which transcriptomes have to be considered. Idem, profiles of UMAP embedding for EGF conditions look like very different. What does it means (no EMT ?...)
See response to previous comment. As mentioned, even the most striking EMT conditions (based on morphology) have as little in common with each other than they do with the seemingly weak responses (**Fig 2d**). As such, focusing on a subset of the samples leads to potential bias towards a specific "type" of EMT and does not meaningfully change the findings of the study.

Specifically regarding the UMAP profiles of the EGF conditions, these conditions often involved a smaller number of dynamic genes. As we only used genes from the psupertime model to build UMAP embeddings, the smaller number of genes makes for noisier embeddings.

Authors chose to take genes that were differentially expressed in at least 8 of their conditions. This cut-off is not explained. The paper is lacking a solid message. The authors proposed a list of 86 genes that they also found in variable cancer cells "as well as healthy epithelium from various mouse tissues". This last sentence is not clear? Not convincing if the genes are found in cancer models and healthy tissues. What is the specificity of these genes? Are these genes associated/correlated with clinical parameters in patients/models (progression free survival, metastasis, migration…)?
May these genes a common regulation (what about transcription factor binding prediction in the promoters? common epigenetic regulation?)
Thank you for letting us know that this wasn't clear. The cutoff of 8 conditions (2/3 of the time course experiments) is an arbitrary cutoff to identify the most conserved genes without being too restrictive. We have added a statement about this decision on **lines 144-145**. Further this yielded a conveniently sized gene list for gene set scoring or enrichment analysis.

As for the relation to human/mouse tissues, we have added clarification on **lines 157-164**. Briefly, assessing these tissues was simply to ensure that the molecular program we identified existed *in vivo* and wasn't simply an experimental artifact. By showing that the genes are variably expressed and well-correlated across

epithelial/carcinoma cells comprising individual tissues (not variable between tissues), it suggests that they contribute to a coordinate heterogeneity program in these samples.

We have added GO term analysis and **lines 148-154** to provide a better description of traits associated with these genes. And thank you for the recommendation of predicting transcription factor regulators. We did this analysis and found an overwhelming enrichment of AP-1, MYC, MEF2, and KLF binding sites in their regulatory regions, suggesting that these may be common regulators across conditions. We have added **Lines 146-157** and **Extended Data Fig 7a,b** to reflect this.

All along the manuscript, it is difficult to understand what is experimentally done without be obliged to constantly refer to Mat & section or tables. (few examples: "EMT time course", "several EMT genes, "variety of effects", "no EMT controls"…) Some details should be added in the main text. Why authors used these 22 inhibitors (does it refers to the 16 molecules written above in the manuscript ?) is not evident to understand.
We apologize for the confusion. In the additional text we have added throughout the manuscript, we have made efforts to be more clear. We have made the details of the time course experiments clearer on **lines 83-86**, and have changed the wording in the abstract to avoid the inclusion of "16" (**lines 15-16**), which referred to the number of screens that were performed (ie. 22 inhibitors x 16 conditions).

The name of the target of the molecule under brackets is confusing. Ex: "LY364947 (TGFBR1)" should be replaced by "LY364947 (TGFBR1i)"
We have made this change on **lines 207-209**.

The authors showed that inhibition of EGF may partially block induction via TGF. This should be better explained. Could the MAPK pathway be involved in this process since MAPK is in a crosstalk between the non smad pathway (TGFBR) and the EGFR pathway. Molecular experiments are needed to support this observation.
We have added a much more comprehensive interpretation of our kinase inhibitor screens (**lines 214-255**). Given relationships between various pathways, we have provided putative mechanistic explanations for the various effects we have seen. Given the large number of combinations we assessed (16 conditions x 22 inhibitors), further interrogation into these pathways is beyond the scope of this specific project. We do, however, believe that these findings warrant further study.

Kinase inhibitors are used concomitantly with EMT inducers. It could be useful to repeat some experiments with a pre-treatment (e.g. one day before) with the inhibitors to analyze whether inhibition of the target kinases could really block EMT.
The temporal relationship between inhibition and EMT induction is absolutely an interesting concept. It certainly seems feasible that some effects may vary depending on the timing of inhibition. That said, we at least believe our approach of concomitant treatment of the inhibitor and EMT inducer is valid based on the ability of TGFBR1 and EGFR inhibitors to completely negate the effects of TGFB1 and EGF (**Fig. 4f,g)**, respectively, even though they were added at the same time. Given the scale of the experiment, assessing different inhibition timings here would be cost prohibitive. These experiments are best suited for follow-up studies of the specific pathways highlighted here.

The specific choice of the OVCA420 cells for the ATAC-seq experiment is not explained
We have added **Line 183-186** to explain this better. OVCA420 + TGFB1 was the smallest dataset in our time course cohort, providing the lowest power for inferring transcription factor activity, making it a good candidate for validation. We emphasize that this was just for validation of the computational method, ensuring that our inference of transcription factor activity could be supported.

Some papers previously reporting data also observed in the present paper should be cited.

Gemmil RM et al 2011 Cancer Lett showing that cancer cells are not epithelial or mesenchymal but frequently between these phenotypes.
Peixoto P et al 2019 Cell Death Dis, showing with transcriptomes in different cell models that gene expression was more closely dependent from the cell line than the EMT induction.
Du L et al Cancer Med, analyzing the signature of TGFb-induced EMT
Cieslik M et al Epigenetics Chromatin showing the links between epigenetics and transcriptomes in EMT
Thank you for bringing these papers to our attention. We have added references to quite a few more papers in the additions to the revised manuscript, including the Gemmil, Peixoto, and Cieslik papers you have mentioned.

Referee #2

Cook et al. offer a comprehensive single cell transcriptomic analysis of EMT that explores a variety of cancer cell lines exposed to different EMT inducers with a time series experiment that also includes a drug inhibition study. Data is generated through multiplexed single cell RNA sequencing. The resulting dataset is rich and will likely serve as a landmark dataset that will be further explored by many others for years to come.

Using the unique data they generated, the authors make several conclusions about EMT. Notably, they find that EMT is a context-dependent process, with transcriptional dependency on the cell line and EMT-inducing factors tested. Four different cell types were probed (A549, lung; DU145, prostate; MCF7, breast; OVCA420, ovarian) and each was separately treated by one of three different EMT-inducing factors (TGFB1, EGF, and TNF). The authors demonstrate that many of the commonly cited EMT Hallmark genes are not differentially expressed after treatment. The authors produce a new EMT gene list that they claim is most invariant to the conditions tested. Their drug screening analysis on the EMT-induced cells with small molecule inhibitors further demonstrates context-dependent effects.

Overall, the study addresses timely and elusive issues in the EMT/cancer field and provides a valuable new dataset as a resource that can be further mined by the broader community. While the authors present the experiments clearly, the take home points derived from the data analytics are overly broad and not unexpected. Indeed, it is already recognized that EMT is a context-dependent and heterogeneous process. The data generated here may be able to quantify the EMT process in an unprecedented manner but the analysis provided is largely descriptive and leaves several important questions unanswered. Listed below are such questions, together with comments that point to potential limitations of the study.

Comment regarding general changes to manuscript text
We would like to thank each of the reviewers for taking time to evaluate our manuscript and for providing thoughtful comments. These comments have inspired a number of additions to the text of the manuscript. While we respond to each specific comment below, the following is an outline of some of the more general changes to each section of the manuscript:

Introduction
We have added much more background and justification for our study. We hope that this does a better job at presenting the key questions we hoped to address.

Results
We have improved the clarity of methodological details, including justification for the conditions we assessed, and relevant references. We have also provided a much more thorough interpretation of our data, particularly regarding our list of conserved EMT genes and the impact of the various kinase inhibitors on EMT progression.

Discussion

This section has been completely rewritten. Interpretations of specific pieces of data are largely contained at relevant points of the results section, so the discussion section is fairly concise, focusing on the broad points that we think are critical to convey.

All modifications are highlighted in the revised manuscript.

1) Given the vast number of EMT studies, including recent single-cell analysis studies (scRNAseq specifically), why do the authors not mention those studies and put their work in that current landscape? Some important and highly relevant studies include Pastushenko et al "Identification of the tumour transition states occurring during EMT", van Dijk et al: "Recovering gene interactions from single-cell data using data diffusion." There are many others.
We have expanded the narrative throughout the manuscript to better put our work in the context of the current literature. In the introduction, we have added more information about studies that have shown variable EMT responses (including several single-cell analyses). We believe we have also structured it to better emphasize the purpose of our own study and the main questions we hoped to address. Throughout the results, we have also related our findings to previous studies. Most of the changes to the manuscript text was for this purpose. Sections are highlighted in the revised manuscript.

2) Given that the data is generated with single cell resolution, there is surprising little to no analysis of cell clusters that may be similar or unique to the various conditions tested. For example, some relevant cell clusters may be the partial EMT states previously reported in the literature. The data that the authors have generated offer the opportunity to explore this possibility and frame the results of this study into a more meaningful context for the broader field.
We believe that the revised manuscript links our findings with the literature much better. With regards to the specific analyses performed, we opted to focus on comparing (pseudo)temporal dynamics of the transition across the variety of conditions we assessed, as we were able to perform them together in a well-controlled design. We chose to use the hallmark EMT gene set as our single reference from the literature, but we certainly could have assessed signatures from other studies. Problematically, there are so many gene sets now that there would have been selection bias. We were most surprised by how dramatically dissimilar each EMT response was in our study, and given these variable responses, we believe that the idea of strict "full" and "partial" EMT signatures is inaccurate. We wanted this to be a key message of the paper and hope that the revised manuscript is convincing of that. A more thorough study of intermediate states is certainly warranted, but this is probably best suited for studies of "naturally occurring" intermediate states rather than experimental time courses of fairly strong EMT induction.

3) The authors claim that the EMT hallmark gene set contains many genes that are not differentially expressed by any of cancer cell lines treated by various EMT-inducing factors. This finding is not surprising as it has been documented that some of the genes in the EMT Hallmarks are derived from stromal cells (i.e. fibroblasts) in the tumor microenvironment that coordinate the mesenchymal transformation of the epithelial malignant cells through paracrine signaling. These stromal cell types are captured in bulk tumor gene expression studies. Many deconvolution efforts are demonstrating the heterogeneity of cell types contributing to bulk-derived gene expression signatures.
We have added **lines 137-139** to highlight this limitation of the Hallmark gene set.

4) The authors provide a new EMT gene list that they claim is most invariant to the conditions tested and more suitable for assessing EMT, despite its context-dependence. <u>However no discussion is provided on the new genes being proposed and why they may be biologically relevant to EMT.</u> This is a limitation of the paper, given that the gene list is a main result of the paper. In addition, it could be instructive to examine if specific genes from their list are altered during the kinase inhibitor treatments. Such a finding could explain the effects of the inhibitors in the context of the authors' main findings.

Thank you for bringing this up. We have expanded this section in the manuscript (**lines 146-157**) to provide a bit more of a "biological explanation" of what these genes are. We also added GO term analysis of the genes and showed that these conserved genes do enrich for terms related to EMT-associated traits (**Extended Data Fig 7a)**, and predict transcription factor regulators of these genes (**Extended Data Fig 7b**). We also emphasize that no individual genes are universal markers and that the list is simply the most conserved changes. As for assessing these genes in the kinase inhibitor experiments, we did explore this and found that genes affected by inhibitors were not preferentially from the list (nor were they significantly depleted from the list). As this finding lacks any meaningful conclusion, we have chosen to not include it in the paper. We have, however, included a much more comprehensive interpretation of the kinase inhibitor data (**lines 214-255**).

5) The definition of EMT across the different conditions seems to be "whatever happens after 7 days under the influence of the EMT inducer" regardless of the cell line and the treatment condition. This limitation is not addressed. From the pictures provided in Extended Data Figure 1, EMT-related morphological changes are not as evident in some cell lines under certain conditions. Do all the cell lines express the receptors of the given ligands? Can references be provided as to whether all these ligands induce EMT in these cell lines? For the MET process, it is not clear that under all conditions all cells reverted after 3 days as stated for Figure 2b. Was there a reason a longer withdrawal time-course was not tested?

Thank you. This is also a great point. We have addressed these comments in several places:

1) On **LInes 68-73**, we provide a better justification for the cell lines we have chosen and have referenced studies (refs. 14-20) that have documented the ability of these inducers to promote an EMT in these cell lines.
2) We have provided a more explicit description of the morphological differences between conditions on **lines 74-76**. We have also included an interesting reference to a study showing that EGF can promote a "circular" morphology along with mesenchymal traits when used at higher doses. We have added **lines 76-81** to speculate why different morphologies can be observed.
3) We have included a plot in **Extended Data Fig 3** showing detectable expression of the ligands' receptors in each cell line.
4) Regarding the MET following stimulus withdrawal, a max time of 3 days was chosen based on preliminary experiments with TGFB1 treatment in OVCA420 cells that showed transcriptional reversion in as early as 3 days (**lines 85-86** have been added to state this), even though morphological changes required longer periods to revert. In our actual experiment for the paper, many of the conditions had near-complete reversion in 3 days (Fig 1d), but this certainly wasn't the case for all conditions. Additional time points weren't added to keep the scale of the experiment reasonable.
5) We have added **lines 111-113** stating the limitation of restricting our time course to 7 days. It would be interesting to perform extended time courses to assess the limits of various EMT responses, but this was not feasible for scaling limitations. Despite this, we do not think that this affects the main conclusions of the paper. Specifically, if cells were to continue transitioning, it does not change that the expression programs associated with each response are very different. While not in this study, we have observed a plateau in the EMT response between 3-7 days in several other experimental systems, suggesting that this may be the length of the response under certain conditions.

6) Are there any data showing that the direct targets of all inhibitors used in Figure 4C are present in these cell lines? Protein levels would be preferable even though the paper focused on RNA. Although all effects are assessed relative to EMT "output", this would shed light on whether some of the results are off target effects. Also, given that the results of Figure 4 are assessed as an EMT dependence on paracrine signaling, it would be helpful to annotate which paracrine signaling is inhibited with each inhibitor. For example, PI3K kinase is involved in many different signaling pathways (not only paracrine), and has been implicated with many of the molecules stated to be inhibited by the drugs tested.

We have added a fair amount of text to this section of the manuscript (**lines 214-255**) to extend the interpretation of these results. In these interpretations, we have been more explicit about the specific targets and pathways affected

by the inhibitors. Note that the kinase targets are also listed in Figure 4c, many of which are expressed fairly ubiquitously expressed in different cell types. This section also includes a more thorough discussion of inhibitors other than Nec-5 (RIPK1i). We also use information from previous studies to suggest that the transcriptional changes of many of the combinations are at least consistent with inhibition of the proper target. This does not preclude the possibility of off-target effects, but if they are present, we suspect they exist in combination with on-target effects, making interpretation of protein analysis challenging (ie. a western blot may show kinase inhibition, but off target effects are still possible). Further, given the large number of conditions to be assessed (ie. 22 different kinase inhibitors in all 16 cell line-inducer combinations), protein analysis of each would be quite extensive. Given all of this, we think that a more mechanistic analysis of the specific pathway mechanisms is beyond the scope of this specific project.

As for RIPK1 specifically, we now provide a putative mechanism in the text (**lines 241-255**), but think that a more-detailed analysis would warrant a separate study. We did look at morphology in the inhibited cells and found that Nec-5 does *not* inhibit the morphological changes associated with TGFB1 treatment. Interestingly, however, some of the genes that are not activated when RIPK1 is inhibited are ECM and mesenchymal-associated adhesion proteins, but others are not affected by Nec-5. As the interpretation of this isn't entirely clear, we have chosen to omit it from the manuscript.

7) Regarding the discussion around the results from RIPK1inhibition, how are EMT and TGFb related to necroptosis? What does blocking cells "half way into EMT" mean? Are the authors referring to a partial EMT state? Are the effects of RIPKI (and some of the other inhibitors) confirmed by EMT-related morphological assessment? Do these drugs inhibit TGFb-induced EMT morphological changes? Are any of these results agreeable with some protein data? EMT can manifest at the level of posttranslational modifications of common markers such as E cadherin, (e.g. Aiello et al. 2018).

1) Necrosome formation has been associated with ERK1/2 activation through RIPK1. ERK1/2 activation can lead to increased activity of the AP1 complex. The genes preferentially blocked by RIPK1 inhibition are AP1 targets. We have added this information to **lines 241-248**.
2) We have used clearer language to replace "half way through the EMT" on **lines 234-239**. We notedan important distinction that the partial response following Nec-5 treatment is not a temporal block (ie. stalling the cells' progression and preventing late EMT dynamics), but rather, it has "modular" inhibition, blocking an independent component of the EMT response, simply preventing some expression dynamics (AP1 signalling, for example), but not others.
3) Regarding morphological changes, see the response to the previous question.
4) While the finding of Aiello *et al*. is interesting, we're not certain that looking at E-cad cellular localization would add to the interpretation or conclusions of our study. I would argue that although this PTM-mediated regulation of the transition may be important, it is unlikely that it is not associated with global gene expression changes. We focus on global expression profiles more as a general read-out of the cells' state rather than to focus on a subset of specific features, allowing for a higher-level comparison of the EMT responses.

Referee #3

The manuscript submitted by Cook and Vanderhyden reports that epithelial-mesenchymal transition (EMT) involves various transcript expression profiles among four carcinoma cell lines exposed to three distinct EMT inducers.
The study is based on single-cell sequencing at different time-points and in the presence of kinase inhibitors. ATAC-seq was performed in one cell line. Cells from each well were barcoded to allow demultiplexing pooled cells from each of the 96-well plate experimental designs.
Bioinformatics approaches were applied for quality controls and for establishing the most variable gene list across the different time-points of the EMT kinetics. A pseudo-temporal ordering is provided for each cell line during EMT progression, spontaneous reversal and after reversal by selected drugs interfering with the

EMT inducer signaling pathways. The ATAC data obtained with the OVCA420 carcinoma cell line showed good concordance with the transcription profiles.

__**Comment regarding general changes to manuscript text**__

We would like to thank each of the reviewers for taking time to evaluate our manuscript and for providing thoughtful comments. These comments have inspired a number of additions to the text of the manuscript. While we respond to each specific comment below, the following is an outline of some of the more general changes to each section of the manuscript:

Introduction

We have added much more background and justification for our study. We hope that this does a better job at presenting the key questions we hoped to address.

Results

We have improved the clarity of methodological details, including justification for the conditions we assessed, and relevant references. We have also provided a much more thorough interpretation of our data, particularly regarding our list of conserved EMT genes and the impact of the various kinase inhibitors on EMT progression.

Discussion

This section has been completely rewritten. Interpretations of specific pieces of data are largely contained at relevant points of the results section, so the discussion section is fairly concise, focusing on the broad points that we think are critical to convey.

All modifications are highlighted in the revised manuscript.

__**Comments**__

It is not clear why, although the authors consider 12 time-points for the growth kinetics, only 7 time-points are described, including 3 time-points for spontaneous reversal.

We apologize for the misunderstanding. There are 12 independent time course experiments, and for each, we assess 8 time points (5 treatment time points, and 3 withdrawal time points). We have made this clearer in the results section of the manuscript (**lines 83-86**)

Only slightly more than half of the 103,999 cells were analyzed.

Again, we apologize for this misunderstanding. The 103,999 cells mentioned in the abstract correspond to the combination of the time course experiments (58,088 cells) and the kinase inhibitor experiments (45,911 cells).

TNF is not usually used as EMT inducer. What motivated the authors to use this cytokine and which TNF have they used?

The general rationale for choosing these EMT inducers (TGFB1, EGF, and TNF alpha) was the desire to assess EMTs driven by largely independent signalling pathways. We have added text to explicitly explain this in the manuscript (**lines 70-73**). While not the most common EMT inducer used, there are numerous papers describing TNF's ability to promote the transition. Other inducers could have been used (eg. certain Wnt ligands), but with the scale of this scRNA-seq study, it was not feasible to add more samples. We have also added references to studies that examined the ability of these inducers to promote the EMT in these specific cell lines (refs. 14-20)

The authors have selected 4 cell lines with very distinct phenotypes and degree of responses to each EMT inducer.

We have added more text to explain the motivation behind choosing these cell lines (**lines 68-73**). We have also added a brief description of the morphological changes observed in each cell line (**lines 73-81**).

Extended Figure 1a shows that A549 lung adenocarcinoma cells exhibit mixed EMT phenotypes, as shown by multiple studies. This cell line is already engaged in the EMT process at baseline; it responds well to the three inducers used in this study, with no obvious cell death. In contrast MCF7—one of the most epithelial-like cell lines of breast carcinoma—is known to be quite refractory to EMT inducers. Extended Figure 1a suggests that very few cells succeed in engaging into EMT only with TNF. A similar comment can be made for the two other cell lines: DU145 is a rather mesenchymal-like cell line that, again, responds differently to the three inducers with quite distinct morphologies. OVCA420 is epithelial-like cell line that exhibits very distinct responses to the three inducers. Again, one can suspect that TGFb1 induces cell death. The cell counts shown in extended Figure1c reflect this issue.
A description of the observed changes to cell morphology has been added (**lines 73-81**). MCF7's morphological changes are certainly subtle, but we do believe they are evident. We believe the morphological changes in response to TNF are clear across cell lines, but we do note the changes are interestingly different from those induced by TGFB1 in the same cell line (mentioned in **lines 74-75**). Regarding the unique morphology of OVCA420 cells in response to TGFB1, we also initially thought that this was associated with cell death. While we don't include experiments here, we have noted that there is only a small amount of death, but the cells' proliferation rate drops dramatically. This is why the cell counts for OVCA420 were so low.

We have also added more analysis of the list of conserved differentially expressed genes and show enrichment for EMT-associated traits (eg. ECM disassembly and organization, cell migration, negative regulation of apoptosis; **lines 148-154, Extended Data Fig. 7a**). This further supports that although some of the morphological changes aren't as striking as others, they all experience changes that are associated with an EMT.

In Figure1d, EGF treatment led to very modest shifts for all 4 cell lines.
Yes, EGF was definitely the most modest inducer of the three. That said, EGF induced many gene expression changes that were still enriched for EMT traits (**Extended Data Fig 5a, Extended Data Fig. 7a**).

The EMT hallmark (Figure1f) shows a similar profile for all conditions. How can this be explained with these very distinct morphologies?
The most likely explanation is that each condition is associated with changes in different subsets of ECM components, proteases, and adhesion proteins. While the GSEA profiles of the EMT hallmarks show that all conditions enrich for the gene set, the specific genes from the set that are most variable are different between data sets. We have added text on **lines 79-81 and lines 135-141.** We also refer to the heatmap of hallmark genes (Extended Data Fig. 6), which shows variable changes in these components across settings.

The EMT score used in this study (Figure 2d) is not significantly increased and, in several cases, does not vary in the pseudo-time analysis.
While 2 of the sample's EMT scores (based on the hallmark gene set, which we show isn't the most reliable collection of EMT-associated genes) do not change over pseudotime, we show in Extended Data Fig 3c that this is due to some EMT hallmark genes being downregulated over pseudotime, resulting in a net neutral EMT score (explained in **lines 120-123**).

The EMT signature derived from the four cell lines was then used to analyze two databases for lung cancer and one database for normal mouse tissues (Extended Figure 4). The graphs do not reveal any significantly better results than other signatures. For instance, it well known that basal cells have EMT features, and that most tumors exhibit at a partial EMT phenotype (see for instance Tan et al. EMBO Mol Med, 2014).
The purpose of exploring the cancer and normal tissue samples was just to ensure that the conserved genes exhibited coordinated variability *in vivo* and weren't just an experimental artifact. For this reason, we confirmed that the genes were overdispersed and well correlated in the scRNA-seq data from these samples. The results suggest that this molecular program also exists *in vivo* (explained in **lines 157-164**). We did not perform any actual scoring using the gene set to determine relative full/partial E/M status.

The authors used 22 small-molecular compounds with variable specificity to induce EMT reversal in the 4 cell lines in the absence and presence of the EMT inducers. Some of these drugs, as expected, dampened the response to the inducers. Not surprisingly, TGFbetaR inhibitor affected EGFR signaling in A549 cells, since TGFbetaR requires receptor tyrosine kinase signaling for activation in a number of cell lines (Derynck and Budi Sci Signal 2019).
TGFB1 ligand also increases throughout the EMT in many of the conditions, so the susceptibility to TGFBR1 inhibition may also be through blocking autocrine/paracrine signalling initiated by various inducers. We have extended the section corresponding to the kinase inhibition experiments to include much more interpretation of the results (**lines 211-255**).

The authors conclude that the expression profiles vary considerably in each condition and that only a subset of well-known EMT genes are revealed in this study. This finding is not surprising due to the choice of these 4 cell lines of different origins with distinct EMT features at baseline and variable response to the inducers. Notably, several EMT expression profiling signatures do not include most of the canonical EMT genes, which are often not highly expressed albeit detected by PCR.
It is possible that the activity of canonical EMT transcription factors may be predictable based on features of the baseline state of the cells, but this is speculative. In the literature, these factors are often claimed to be directly correlated with E/M status (eg. Zeb1-mir200 axis). Our experiments take cells at some basal state along an E/M spectrum, experimentally induce them to become more mesenchymal, and fail to see changes associated with these factors. Given the assumptions in the literature mentioned above, we believe this is noteworthy. Detection rate due to low expression contributes to this in some cases, but other factors are reliably detected, but still invariable.

Studies of EMT in development show very distinct signaling systems in gastrulation and in subsequent EMT events in morphogenesis and organogenesis. In cancer, it is also apparent that different tumor types will utilize different signaling pathways to gain intermediate EMT stages. There is also increased evidence for intratumoral heterogeneity for the EMT spectrum in individual cancer cells. The authors should consider these concepts in their Discussion.
Yes, absolutely. Our data is in agreement with this notion of variable signalling networks across contexts, and believe this idea is better represented in the revised manuscript. We have added a much more comprehensive discussion and interpretation of the kinase inhibitor screens (**lines 211-255**), highlighting different patterns of different signalling dependencies. The analysis of RIPK1-inhibited cells (**lines 234-239**) also shows that these pathways can contribute to independent components of the EMT, suggesting that a single response can comprise modular expression programs.

REVIEWERS' COMMENTS:

Reviewer #3 (Remarks to the Author):

The authors have provided a rebuttal solely based on rewording some paragraphs. Although they have somewhat clarified some of the criticisms raised by the reviewer, it is clear that the present study is facing the same issues regarding the heterogeneity of response in part due to the extent to which each cell line can engage into EMT.
Contrary to what the authors claim (lines 125-128), it is not surprising that whatever the EMT inducer used in the study, each cell line transcript will always cluster together with the parental cell line, since each cell line has a strong gene expression profile linked to its tissue of origin.
It is also worrisome that the authors have not detected at least Zeb 1 in A549 cells perhaps due to the real limitations of the single-cell sequencing technology. The relatively recent technology still suffers from several issues in terms of detecting a large fraction of transcripts, even in cell lines. Another intrinsic difficulty in single-cell sequencing is that cells are not synchronized and are analyzed at different positions of the cell cycle, thus increasing the variability in the transcriptomes. The authors should have carried out RNA sequencing for each sample to serve as a reference and to determine whether they should have detected important transcripts, including EMT transcription factors, in their single-cell sequencing.
The gene signature derived from this study is of no surprise, since the most expressed genes are related to the extracellular matrix and proteases known to be common denominators for all cell lines and tumors engaged in EMT. However, in tumors, these genes are mostly expressed by stromal cells. This new signature needs to be evaluated in tumors and in cell lines in large datasets to evaluate its performance relative to other signatures.
The drug screen, although potentially interesting, is quite preliminary, as stated by the authors. It has not delivered interesting data leading to the identification of potential new targets, aside from RIPK1, which can contribute to activation of some EMT modules.

Reviewer #4 (Remarks to the Author):

Through multiplexed single cell RNA sequencing, the study by Cook et al., provides a comprehensive, rich EMT dataset that will be undoubtedly utilized by the broader EMT community for cancer related studies. The authors have adequately addressed the concerns that had been raised through clarifications in the text, detailed discussion and adding references and requested supplementary data.
Although the study is strong in its approach and analytics,and offers in depth views of EMT dynamics - all valuable for future studies - I do feel that the main message, that EMT is a context-dependent and heterogeneous process, is perhaps not as novel and is already recognized in the field.

Referee #3 - Second round of comments

The authors have provided a rebuttal solely based on rewording some paragraphs. Although they have somewhat clarified some of the criticisms raised by the reviewer, it is clear that the present study is facing the same issues regarding the heterogeneity of response in part due to the extent to which each cell line can engage into EMT.
We believe that one of the main conclusions of our study is that the notion of EMT “extent” is undefinable. Consider A549 and DU145 cells exposed to TGFB1. Both undergo a very clear morphological transition, and yet only share ~20-25% of their differentially expressed genes in common. If there were a common EMT program, but different cell types exhibited different degrees of the response, we would still expect to see higher overlap. For example, perhaps all cell lines would have the same early response genes, but only certain cell lines would successfully activate a late EMT program. While we certainly agree that some of the responses are more robust than others, this does not sufficiently explain the variability we see in our data.

Contrary to what the authors claim (lines 125-128), it is not surprising that whatever the EMT inducer used in the study, each cell line transcript will always cluster together with the parental cell line, since each cell line has a strong gene expression profile linked to its tissue of origin.
We would like to note that the analysis referred to here is the Jaccard index of differentially expressed genes in response to EMT inducers, not the cells’ global expression profiles. Yes, similar global expression profiles would be expected due to common tissue of origin, mutational profile, etc. The Jaccard index presented in the manuscript refers to the proportion of differentially expressed genes that are common between conditions. The field frequently generalizes the effect of different signalling pathways across cell types. Therefore, we maintain that it is surprising that different growth factors/cytokines—each activating different signalling pathways—promote more-similar responses in an individual cell line than a single factor does in different cell lines.

It is also worrisome that the authors have not detected at least Zeb 1 in A549 cells perhaps due to the real limitations of the single-cell sequencing technology.
In the manuscript, we made no claims specifically about the detection of Zeb1, or any of the transcription factors, in the cell lines. We focused on comparing the genes that actually change throughout the response. We certainly recognize that we did not comment on the detection of various factors. As such, we have added **Extended Data Fig. 9** and **lines 173-176** to show that in many cases, the hallmark EMT transcription factors were detected, but not significantly changed through the EMT. Zeb1 was readily detected in A549 cells, but did not show significant temporal dynamics.

The relatively recent technology still suffers from several issues in terms of detecting a large fraction of transcripts, even in cell lines.
Regarding assay sensitivity, our added **lines 173-176** acknowledge that failed detection may be due to a somewhat low assay sensitivity. That said, our scRNA-seq libraries are relatively complex and adequately sequenced: for the time course data, we detected a median of 3649 genes and 17,330 unique molecules per cell. We have included these statistics in our methods section on **lines 351-353.** Common with scRNA-seq data, individual cells may lack detection of specific genes, each cell profile in the data represents a different subsample of the cells’ complete transcriptome. By sampling dozens-to-hundreds of individual cells from the population, the aggregate expression profile is quite representative of the complete transcriptional profile.

Another intrinsic difficulty in single-cell sequencing is that cells are not synchronized and are analyzed at different positions of the cell cycle, thus increasing the variability in the transcriptomes.
Confounding effects of cell cycle-associated gene expression can confound all expression analysis — not just scRNA-seq. A benefit of single-cell analysis though, is that the cell cycle phase of every individual cell can be inferred and dealt with in whichever way is deemed most appropriate. When dealing with data from relatively pure populations of cells, cell cycle is a major source of gene expression variation. As we mention in **Lines 379-383**, we calculated cell cycle scores for every cell and regressed out cell cycle effects from the gene expression data prior to

dimensionality reduction. As a result, cell cycle-associated variation does not contribute to our UMAP projections or pseudotime embeddings.

The authors should have carried out RNA sequencing for each sample to serve as a reference and to determine whether they should have detected important transcripts, including EMT transcription factors, in their single-cell sequencing.
Average expression profiles from libraries generated on the 10x Genomics Chromium platform correlate strongly (pearson correlation >0.9) with bulk RNA-seq profiles (see supplemental figure 10 in Ding *et al., BioRxiv*, 2019, https://doi.org/10.1101/632216)). Further, as mentioned above, our libraries were fairly complex, with thousands of genes detected per cell and over half of the transcriptome reliably detected across the whole population of cells. And finally, many of the EMT transcription factors were readily detected in our data, but were not differentially expressed. Therefore, we would argue that performing bulk RNA-seq as a reference would not provide additional benefit.

The gene signature derived from this study is of no surprise, since the most expressed genes are related to the extracellular matrix and proteases known to be common denominators for all cell lines and tumors engaged in EMT. However, in tumors, these genes are mostly expressed by stromal cells. This new signature needs to be evaluated in tumors and in cell lines in large datasets to evaluate its performance relative to other signatures.
While the classes (eg. ECM and cell adhesion proteins, proteases) may have been predictable, many of the genes comprising the signature are not commonly considered EMT genes. Further many of the common EMT genes are not represented in our gene list. We explored the expression of this gene list in various tumour cells and epithelial tissues (Supplemental Figure 8) and found that the genes are detectable and their expression is well-correlated across individuals of the given tissue, suggesting that the signature may be a conserved gene set associated with E-M plasticity *in vivo.*

The drug screen, although potentially interesting, is quite preliminary, as stated by the authors. It has not delivered interesting data leading to the identification of potential new targets, aside from RIPK1, which can contribute to activation of some EMT modules.
In our view, the drug screen provided three intriguing conclusions: 1) RIPK1 had not been previously implicated in the EMT, and here we found it important for several EMT responses, 2) consistent with several focused studies, ERK1/2 and AP-1 activity appear to be common effectors of many EMT responses, and 3) the EMT response is modular and it seems that components of it can be inhibited without affecting other dynamics. The final point is important given that much of the field depicts the response as a single continuum, whereas data is more consistent with the transition involving multiple independent components.

Reviewer #4

Through multiplexed single cell RNA sequencing, the study by Cook et al., provides a comprehensive, rich EMT dataset that will be undoubtedly utilized by the broader EMT community for cancer related studies. The authors have adequately addressed the concerns that had been raised through clarifications in the text, detailed discussion and adding references and requested supplementary data.
Although the study is strong in its approach and analytics,and offers in depth views of EMT dynamics - all valuable for future studies - I do feel that the main message, that EMT is a context-dependent and heterogeneous process, is perhaps not as novel and is already recognized in the field.
Thank you for taking the time to review our work and for the compliments regarding the approach and analytics. We recognize that many have appreciated some degree of context specificity (eg. developmental EMTs, wound healing, metastasis), but EMT literature fails to capture how variable the response can be: many studies still draw broad conclusions from the expression of a small number of “EMT genes”, review articles describe “core regulatory

networks" generally associated with the EMT, the transition is still often depicted as a one-dimensional continuum with defined traits at certain points along its length, etc.

We believe our study offers several novel insights for the field:

1) We directly assess and quantify the extent of EMT variability in controlled experiments. In our study, only ~20-25% of genes involved in the EMT response are shared between conditions, and cell type is a better predictor of the response than the specific factor inducing the response.
2) Hallmark EMT genes (eg. EMT transcription factors, E-cad, N-cad) are relatively poor markers of the EMT response. Related, the idea of "core regulatory machinery" comprising the classic EMT transcription factors is, at best, an oversimplification. Other transcription factors typically not associated with the EMT (eg. SOX4 and AP-1) are more conserved than classic TFs.
3) The EMT response is modular, and components of the response can be blocked without affecting others. Therefore the model of a single response continuum is incorrect, and this modularity likely explains how EMT responses can be so variable. The extent of this modularity will be unclear until further perturbation screens can be performed.
